# Fast interior point solution of quadratic programming problems arising from PDE-constrained optimization

**DOI:** 10.1007/s00211-017-0892-8

**Published:** 2017-05-22

**Authors:** John W. Pearson, Jacek Gondzio

**Affiliations:** 1 School of Mathematics, Statistics and Actuarial Science, University of Kent, Sibson Building, Parkwood Road, Canterbury, CT2 7FS UK; 2 School of Mathematics, The University of Edinburgh, James Clerk Maxwell Building, The King’s Buildings, Peter Guthrie Tait Road, Edinburgh, EH9 3FD UK; 3 NASK Research Institute, Kolska 12, 01-045 Warsaw, Poland

**Keywords:** 65F08, 65F10, 65F50, 76D55, 93C20

## Abstract

Interior point methods provide an attractive class of approaches for solving linear, quadratic and nonlinear programming problems, due to their excellent efficiency and wide applicability. In this paper, we consider PDE-constrained optimization problems with bound constraints on the state and control variables, and their representation on the discrete level as quadratic programming problems. To tackle complex problems and achieve high accuracy in the solution, one is required to solve matrix systems of huge scale resulting from Newton iteration, and hence fast and robust methods for these systems are required. We present preconditioned iterative techniques for solving a number of these problems using Krylov subspace methods, considering in what circumstances one may predict rapid convergence of the solvers in theory, as well as the solutions observed from practical computations.

## Introduction

We are concerned with optimization problems which involve partial differential equations. Problems of this type appear for example in numerous applications of optimal control, where one wishes state variables to be close to a certain desired form and hopes to achieve it by an appropriate choice of control variables. Let $\varOmega \subset {\mathbb {R}}^{d}$, $d\in \{2,3\}$, be a bounded open domain with sufficiently smooth boundary $\partial \varOmega$. An optimal control problem with constraints may be written as:

1 $$\begin{aligned} \min _{y \in Y,~u \in U}~~~{\mathcal {J}}(y,u) \quad \text {s.t.} \quad c(y,u) = 0, \end{aligned}$$

where the state *y* and control *u* belong to appropriate function spaces *Y* and *U*, respectively. The objective ${\mathcal {J}} : Y \times U \mapsto {\mathbb {R}}$ and the constraints $c : Y \times U \mapsto \varLambda$, where $\varLambda$ is another function space, are assumed to satisfy certain smoothness conditions to guarantee the existence and uniqueness of the solution. Many real-life problems may be modelled as optimal control problems (1). There exists rich literature on the subject which addresses specific applications and provides theoretical background to such problems. The rigorous analysis of optimal control problems requires the use of nontrivial function spaces and involves sophisticated techniques from functional analysis. We refer the interested reader to excellent books on the subject [22, 24, 45], while for simplicity in this paper we assume that *Y*, *U* and $\varLambda$ are all equal to ${L_{2}(\varOmega )}$.

The objective function ${\mathcal {J}}$ may take many different forms but it is often given as:

2 $$\begin{aligned} {\mathcal {J}}(y,u) = \frac{1}{2} \Vert y - {\widehat{y}} \Vert _{L_{2}(\varOmega )}^2 + \frac{\beta }{2} \Vert u \Vert _{L_{2}(\varOmega )}^2, \end{aligned}$$

which corresponds to balancing between two goals: keeping the state *y* close to a certain desired form ${\widehat{y}}$, and minimizing the “energy” of the applied control *u*. The constraints *c* in (1) involve some PDE operator(s), and restrict *y* and *u* to $\varOmega$ and its boundary $\partial \varOmega$. Additionally they may include simple bounds on *y* and *u*. In Sect. 3 we will introduce two particular classes of optimal control problems: time-invariant and time-dependent PDE-constrained problems.

Computational techniques for PDE-constrained optimal control problems involve a discretization of the underlying PDE. There are two options for doing this, and the typical paradigm in PDE-constrained optimization literature is for both approaches to solve the problem in a similar manner. The first is to apply an *optimize-then-discretize* method, involving constructing continuous optimality conditions, and then discretizing these. However we find that this approach is inconvenient when examining the resulting discrete systems for the problems considered in this paper, specifically with regard to the reduction of the dimension of the system, as well as symmetry of the matrix involved. The alternative method, which we apply in this paper, is the *discretize-then-optimize* approach: here a discrete cost functional is constructed and discretized constraints are formulated. Then optimality conditions are derived for such (possibly huge) problems. Our motivation for using this approach originates from an observation that for a particular (quadratic) cost functional (2) the discretized PDE-constrained problem takes the form of a quadratic optimization problem for linear PDEs. The use of fine discretization leads to a substantial size of the resulting optimization problem. Therefore we will apply an interior point algorithm to solve it.

Interior point methods (IPMs) are very well-suited to solving quadratic optimization problems and they excel when sizes of problems grow large [17, 52], which makes them perfect candidates for discretized PDE-constrained optimal control problems. The use of IPMs in PDE-constrained optimization is not new. There have been several developments which address theoretical aspects, including the functional analysis viewpoint, and study the convergence properties of an interior point algorithm [46, 49, 51], and many others which focus on the practical (computational) aspects. IPMs belong to a broad class of methods which rely on the use of Newton methods to compute optimizing directions. There have been several successful attempts to use Newton-based approaches in the PDE-constrained optimization context [4, 5, 25, 28]. The main computational challenge in these approaches is the solution of the linear system which determines the Newton direction. For fine PDE discretizations such systems quickly get very large. Additionally, when IPMs are applied, the added interior point diagonal scaling matrices degrade the conditioning of such systems [17] and make them numerically challenging. Direct methods for sparse linear algebra [10] can handle the ill-conditioning well but struggle with excessive memory requirements when problems get larger. Inexact interior point methods [16, 18, 50] overcome this difficulty by employing iterative methods to solve the Newton equations.

Because of the unavoidable ill-conditioning of these equations the success of any iterative scheme for their solution depends on the ability to design efficient *preconditioners* which can improve spectral properties of linear systems. The development of such preconditioners is a very active research area. Preconditioners for IPMs in PDE-constrained optimization exploit the vast experience gathered for saddle point systems [2], but face an extra difficulty originating from the presence of IPM scaling. There have already been several successful attempts to design preconditioners for such systems, see [1, 3, 18] and the references therein.

In this paper, we propose a general methodology to design efficient preconditioners for such systems. Our approach is derived from the *matching strategy* originally developed for a particular Poisson control problem [37]. We adapt it to much more challenging circumstances of saddle point systems arising in IPMs applied to solve the PDE-constrained optimal control problems. We briefly comment on the enjoyable spectral properties of the preconditioned system, and provide computational results to demonstrate that they work well in practice.

This paper is structured as follows. In Sect. 2 we briefly recall a few basic facts about interior point methods for quadratic programming. In Sect. 3 we demonstrate how IPMs can be applied to PDE-constrained optimization problems. In Sect. 4 we introduce the preconditioners proposed for problems originating from optimal control. We consider separately two different cases of time-independent and time-dependent problems. In Sect. 5 we illustrate our findings with computational results and, finally, in Sect. 6 we give our conclusions.

## Interior point methods for quadratic programming

Within this paper, we are interested in the solution of *quadratic programming* (QP) problems. In their most basic form, such problems may be written as3We consider the case where $A\in {\mathbb {R}}^{m\times {}n}$ ($m\le {}n$) has full row rank, $Q\in {\mathbb {R}}^{n\times {}n}$ is positive semidefinite, ${\mathbf {x}},{\mathbf {c}}\in {\mathbb {R}}^{n}$, and $\mathbf {b}\in {\mathbb {R}}^{m}$. This formulation is frequently considered alongside its *dual problem*
where ${\mathbf {z}}\in {\mathbb {R}}^{n}$, and ${\mathbf {y}}\in {\mathbb {R}}^{m}$. We note that a subset of this setup is that of linear programming (LP) problems, where $Q=0$.

In this manuscript, we consider the solution of quadratic programming problems using interior point methods [17]. The nonnegativity constraints ${\mathbf {x}}\ge {}\mathbf {0}$ are “replaced” with the logarithmic barrier penalty function, and the Lagrangian associated with the barrier subproblem is formed:

$$\begin{aligned} {\mathcal {L}}_{\mu }({\mathbf {x}},{\mathbf {y}}\,)={\mathbf {c}}^{\top }{\mathbf {x}} +\frac{1}{2}{\mathbf {x}}^{\top }Q{\mathbf {x}}+{\mathbf {y}}^{\top }(\mathbf {b} -A{\mathbf {x}})-\mu \sum _{j}\log (x_{j}). \end{aligned}$$

Differentiating ${\mathcal {L}}_{\mu }$ with respect to ${\mathbf {x}}$ and ${\mathbf {y}}$ and defining $z_j = \mu / x_j,~\forall j$, gives the *first order optimality conditions* (or *Karush-Kuhn-Tucker conditions*):

4 $$\begin{aligned} A{\mathbf {x}}={}&\mathbf {b}, \nonumber \\ A^{\top }{\mathbf {y}}+{\mathbf {z}}-Q{\mathbf {x}}={}&{\mathbf {c}}, \nonumber \\ x_{j}z_{j}={}&\mu ,\quad {}j=1,2,\ldots ,n, \nonumber \\ ({\mathbf {x}},{\mathbf {z}})\ge {}&0, \end{aligned}$$

in which the standard complementarity condition for (3), that is $x_{j}z_{j}=0,~\forall j$, is replaced with the perturbed complementarity condition $x_{j}z_{j}=\mu ,~\forall j$. IPMs drive the barrier term $\mu$ to zero and gradually reveal the activity of the primal variables $x_{j}$ and dual slacks $z_{j}$. This is achieved by applying Newton’s method to the system of (mildly) nonlinear equations (4)

5 $$\begin{aligned} \left[ \begin{array}{c@{\quad }c@{\quad }c} -Q &{} A^{\top } &{} I \\ A &{} 0 &{} 0 \\ Z &{} 0 &{} X \\ \end{array}\right] \left[ \begin{array}{c} {\varvec{\delta }}{\mathbf {x}} \\ {\varvec{\delta }}{\mathbf {y}} \\ {\varvec{\delta }}{\mathbf {z}} \\ \end{array}\right] = \left[ \begin{array}{c} \varvec{\xi }_{d} \\ \varvec{\xi }_{p} \\ \varvec{\xi }_{c} \\ \end{array}\right] , \end{aligned}$$

where ${\varvec{\delta }}{\mathbf {x}}$, ${\varvec{\delta }}{\mathbf {y}}$ and ${\varvec{\delta }}{\mathbf {z}}$ denote Newton directions, $\varvec{\xi }_{p}$, $\varvec{\xi }_{d}$ and $\varvec{\xi }_{c}$ denote primal and dual infeasibilities and the violation of complementarity conditions. *X* and *Z* denote diagonal matrices with elements of ${\mathbf {x}}$ and ${\mathbf {z}}$ spread on the diagonals, respectively. By eliminating ${\varvec{\delta }}{\mathbf {z}}$, the Newton system (5) is further reduced to a saddle point form

6 $$\begin{aligned} \left[ \begin{array}{c@{\quad }c} -Q - X^{-1} Z &{} A^{\top } \\ A &{} 0 \\ \end{array}\right] \left[ \begin{array}{c} {\varvec{\delta }}{\mathbf {x}} \\ {\varvec{\delta }}{\mathbf {y}} \\ \end{array}\right] = \left[ \begin{array}{c} \varvec{\xi }_{d} - X^{-1} \varvec{\xi }_{c} \\ \varvec{\xi }_{p} \\ \end{array}\right] . \end{aligned}$$

Since for any $j = 1,2,\ldots ,n$ at least one of the variables $x_{j}$ and $z_{j}$ reaches zero at optimality, the elements of the diagonal scaling matrix $X^{-1} Z$ added to the (1, 1)-block may significantly differ in magnitude: some of them go to zero while the others go to infinity. This feature of IPMs [17] is a challenge for any linear equation solver applied to (6). We skip further details about IPMs and refer the interested reader to [17, 52]. We also highlight that ${\mathbf {y}}$ in this description relates to a dual variable, whereas for PDE-constrained optimization the function *y* corresponds to a primal variable—we elect to use the standard notation within the respective fields.

However, before moving on to PDE-constrained optimization, it is worth drawing the reader’s attention to the fact that, although in (3) we assume only the one-sided bound ${\mathbf {x}} \ge \mathbf {0}$, IPMs can also be easily applied to variables with two-sided bounds:

$$\begin{aligned} {\mathbf {x}}_{a} \le {\mathbf {x}} \le {\mathbf {x}}_{b}. \end{aligned}$$

This requires introducing two nonnegative Lagrange multipliers associated with two inequalities. Later on we will denote them as ${\mathbf {z}}_{a}$ and ${\mathbf {z}}_{b}$, respectively.

## PDE-constrained optimization

We now wish to demonstrate how interior point methods may be applied to PDE-constrained optimization problems. These are a crucial class of problems which may be used to model a range of applications in science and industry, for example fluid flow, chemical and biological processes, shape optimization, imaging problems, and mathematical finance, to name but a few. However the problems are often of complex structure, and sophisticated techniques are frequently required to achieve accurate solutions for the models being considered. We recommend the works [22, 45], which provide an excellent introduction to the field.

Let us first consider a time-independent linear PDE-constrained optimization problem with additional bound constraints:7Here *y*, ${\widehat{y}}$, *u* denote the *state*, *desired state* and *control variables*, with ${\mathcal {L}}$ some PDE operator, and $\beta$ a positive *regularization parameter*. The problem is solved on domain $\varOmega$ (with boundary $\partial \varOmega$), for given functions *f*, $y_{a}$, $y_{b}$, $u_{a}$, $u_{b}$.

We will now apply the discretize-then-optimize approach to (7), commencing with the construction of a Lagrangian on the discrete space. The alternative optimize-then-discretize method will guarantee an accurate solution of the continuous first order optimality conditions, however when applied in conjunction with interior point methods the resulting matrix systems are not necessarily symmetric, nor can they be reduced to such low dimensions for these problems as the matrix systems illustrated later in this section. For these reasons, we find it is advantageous to apply the discretize-then-optimize approach for the interior point solution of PDE-constrained optimization problems—we highlight that this follows the approach used in important literature on the field such as [5, 28]. Provided reasonable choices are made for the discretization of the problem, it is frequently observed that both methods lead to very similar behaviour in the solutions, and indeed this paradigm has recently been used to derive discretization schemes for PDE-constrained optimization (see [20], for instance).

We wish to construct a finite element discretization of the cost functional in (7): for the problems considered in this paper it is beneficial to use equal order finite elements for state and control variables, and observe that a discretized approximation of the cost functional iswhere ${\mathbf {y}}$, ${\mathbf {u}}$ are the discretized versions of *y*, *u*. The (symmetric) finite element *mass matrix* *M* contains entries of the form $[M]_{ij}=\int _{\varOmega }\phi _{i}\phi _{j}~\mathrm{d}\varOmega$, where $\left\{ \phi _{i}\right\}$ are the finite element basis functions used, and ${\mathbf {y}}_{d}$ contains entries of the form $\int _{\varOmega }{\widehat{y}}\phi _{i}~\mathrm{d}\varOmega$.

We therefore write (7) on the discrete level as8with ${\mathbf {f}}$, ${\mathbf {y}}_{a}$, ${\mathbf {y}}_{b}$, ${\mathbf {u}}_{a}$, ${\mathbf {u}}_{b}$ the discrete versions of *f*, $y_{a}$, $y_{b}$, $u_{a}$, $u_{b}$. The matrix *K* depends on the PDE operator ${\mathcal {L}}$ considered: for example when a Poisson control problem (with ${\mathcal {L}}=-\nabla ^{2}$) is examined, *K* denotes a finite element *stiffness matrix* with entries $[K]_{ij}=\int _{\varOmega }\nabla \phi _{i}\cdot \nabla \phi _{j}~\mathrm{d}\varOmega$. Alternatively for convection-diffusion control problems (with ${\mathcal {L}}=-\nu \nabla ^{2}+(\mathbf {w}\cdot \nabla )$, and without stabilization applied within the solution method), *K* contains a sum of diffusion and convection terms with $[K]_{ij}=\int _{\varOmega }\big (\nu \nabla \phi _{i}\cdot \nabla \phi _{j}+(\mathbf {w}\cdot \nabla \phi _{j})\phi _{i}\big )~\mathrm{d}\varOmega$.

We observe that, using our equal order finite element method, the matrices $M,K\in {\mathbb {R}}^{N\times {}N}$, where *N* denotes the number of finite element nodes used, and furthermore that ${\mathbf {y}},{\mathbf {u}}\in {\mathbb {R}}^{N}$.

It can be easily seen that the problem statement (8) is in the form of the quadratic programming problem (3), with

$$\begin{aligned} {\mathbf {x}}= & {} \left[ \begin{array}{c} {\mathbf {y}} \\ {\mathbf {u}} \\ \end{array}\right] ,\quad {}Q=\left[ \begin{array}{c@{\quad }c} M &{} 0 \\ 0 &{} \beta {}M \\ \end{array}\right] ,\quad {}A=\left[ \begin{array}{c@{\quad }c} K &{} -M \\ \end{array}\right] , \\ \ {\mathbf {c}}= & {} \left[ \begin{array}{c} -{\mathbf {y}}_{d} \\ \mathbf {0} \\ \end{array}\right] ,\quad {\mathbf {x}}_{a}=\left[ \begin{array}{c} {\mathbf {y}}_{a} \\ {\mathbf {u}}_{a} \\ \end{array}\right] ,\quad {\mathbf {x}}_{b}=\left[ \begin{array}{c} {\mathbf {y}}_{b} \\ {\mathbf {u}}_{b} \\ \end{array}\right] . \end{aligned}$$

It should be highlighted that, as there has been relatively little previous research on interior point methods for PDE-constrained optimization, there are a number of theoretical considerations that one should account for. As discussed in the paper [46], the majority of the theory available for primal-dual interior point methods is based on finite-dimensional mathematical programming, as opposed to the function space setting of optimal control problems. The authors then proceed to carry out a global and local convergence analysis in the $L^{\infty }$ and $L^{q}$ (for $q<\infty$) settings. It is also important to note that the regularity properties of the optimal state and control are different, which as highlighted in [5] is a crucial feature of the continuous (infinite dimensional) problem which tends to be overlooked when moving to a discretized setting. It is essential to recognise the differences between the continuous formulations involving control constraints and state constraints [5, 46], in particular the greater scope for a rigorous analysis of the control constrained problem, as well as the possibility of generating provably mesh-independent algorithms (including interior point methods) for problems with control constraints, in constrast to problems with state constraints [5]. As the main objective of this paper is to demonstrate the possibility of solving large scale linear systems that arise from interior point methods, we focus for the most part on the challenges faced on the discrete level, however it is crucial to also be aware of the issues present when examining the associated infinite dimensional problem, and in particular the implications of the discretization strategy employed.

In the next section we consider interior point methods for solving problems of structure (8), for a range of operators ${\mathcal {L}}$ and all $\beta >0$. Although there has at this point been relatively little research into such strategies, we highlight that the paper [46] considers the numerical solution of problems of this type with control constraints only, and [1] derives effective preconditioners for large values of $\beta$ and ${\mathcal {L}}y=-\nabla ^{2}y+y$. We also point to the development of solvers of different forms to those presented in this paper: in [18] reduced-space preconditioners are considered for optimal control problems, and in [9] multigrid methods are discussed for a class of control problems.

### Newton iteration

We now wish to derive the equations arising from a Newton iteration applied to the (nonlinear) problem (7). Let us define

$$\begin{aligned} {\varvec{{\mathcal {J}}}}\big ({\mathbf {y}},{\mathbf {u}}\big )=\frac{1}{2} {\mathbf {y}}^{\top }M{\mathbf {y}}-{\mathbf {y}}_{d}^{\top }{\mathbf {y}} +\frac{\beta }{2}{\mathbf {u}}^{\top }M{\mathbf {u}} \end{aligned}$$

to be the discrete functional which we wish to minimize. Applying the discretized version of the PDE constraint, alongside a barrier function for the bound constraints as in the previous section, leads to the Lagrangian

$$\begin{aligned} {\mathcal {L}}_{\mu }\big ({\mathbf {y}},{\mathbf {u}},{\varvec{\lambda }}\big )= & {} {\varvec{{\mathcal {J}}}}\big ({\mathbf {y}},{\mathbf {u}}\big ) +{\varvec{\lambda }}^{\top }(K{\mathbf {y}}-M{\mathbf {u}}-{\mathbf {f}}) \\&-\mu \sum _{j}\log \big (y_{j}-y_{a,j}\big )-\mu \sum _{j}\log \big (y_{b,j}-y_{j}\big ) \\&-\mu \sum _{j}\log \big (u_{j}-u_{a,j}\big ) -\mu \sum _{j}\log \big (u_{b,j}-u_{j}\big ), \end{aligned}$$

of which we wish to find the stationary point(s). Here ${\varvec{\lambda }}$ denotes the discretized *adjoint variable* (or *Lagrange multiplier*), $y_{j}$, $y_{a,j}$, $y_{b,j}$, $u_{j}$, $u_{a,j}$, $u_{b,j}$ denote the *j*-th entries of ${\mathbf {y}}$, ${\mathbf {y}}_{a}$, ${\mathbf {y}}_{b}$, ${\mathbf {u}}$, ${\mathbf {u}}_{a}$, ${\mathbf {u}}_{b}$, and $\mu$ is the *barrier parameter* used.

Differentiating ${\mathcal {L}}_{\mu }$ with respect to ${\mathbf {y}}$, ${\mathbf {u}}$ and ${\varvec{\lambda }}$ gives the *first order optimality conditions* (or *Karush-Kuhn-Tucker conditions*):

9 $$\begin{aligned}&\displaystyle M{\mathbf {y}}-{\mathbf {y}}_{d}+K^{\top }{\varvec{\lambda }} -{\mathbf {z}}_{y,a}+{\mathbf {z}}_{y,b}={}\mathbf {0},\end{aligned}$$

10 $$\begin{aligned}&\displaystyle \beta {}M{\mathbf {u}}-M{\varvec{\lambda }}-{\mathbf {z}}_{u,a} +{\mathbf {z}}_{u,b}={}\mathbf {0}, \end{aligned}$$

11 $$\begin{aligned}&\displaystyle K{\mathbf {y}}-M{\mathbf {u}}-{\mathbf {f}}={}\mathbf {0}, \end{aligned}$$

where the *j*-th entries of ${\mathbf {z}}_{y,a}$, ${\mathbf {z}}_{y,b}$, ${\mathbf {z}}_{u,a}$, ${\mathbf {z}}_{u,b}$ are defined as follows:

12 $$\begin{aligned} \left( {\mathbf {z}}_{y,a}\right) _{j}= & {} \frac{\mu }{y_{j}-y_{a,j}}, \quad \left( {\mathbf {z}}_{y,b}\right) _{j}=\frac{\mu }{y_{b,j}-y_{j}},\nonumber \\ \quad \left( {\mathbf {z}}_{u,a}\right) _{j}= & {} \frac{\mu }{u_{j}-u_{a,j}}, \quad \left( {\mathbf {z}}_{u,b}\right) _{j}=\frac{\mu }{u_{b,j}-u_{j}}. \end{aligned}$$

Note that, by construction, the following bound constraints apply for the Lagrange multipliers enforcing the constraints on *y* and *u*:

$$\begin{aligned} {\mathbf {z}}_{y,a}\ge \mathbf {0},\quad \quad {\mathbf {z}}_{y,b}\ge \mathbf {0}, \quad \quad {\mathbf {z}}_{u,a}\ge \mathbf {0},\quad \quad {\mathbf {z}}_{u,b}\ge \mathbf {0}. \end{aligned}$$

Applying a Newton iteration to (9)–(12) gives, at each Newton step,

13 $$\begin{aligned} M{\varvec{\delta }}{\mathbf {y}}+K^{\top }\varvec{\delta \lambda }-{\varvec{\delta }}{\mathbf {z}}_{y,a}+{\varvec{\delta }}{\mathbf {z}}_{y,b}={}&{\mathbf {y}}_{d}-M{\mathbf {y}}^{*}-K^{\top }{\varvec{\lambda }}^{*}+{\mathbf {z}}_{y,a}^{*}-{\mathbf {z}}_{y,b}^{*}, \end{aligned}$$

14 $$\begin{aligned} \beta {}M{\varvec{\delta }}{\mathbf {u}}-M\varvec{\delta \lambda }-{\varvec{\delta }}{\mathbf {z}}_{u,a}+{\varvec{\delta }}{\mathbf {z}}_{u,b}={}&-\beta {}M{\mathbf {u}}^{*}+M{\varvec{\lambda }}^{*}+{\mathbf {z}}_{u,a}^{*}-{\mathbf {z}}_{u,b}^{*}, \end{aligned}$$

15 $$\begin{aligned} K{\varvec{\delta }}{\mathbf {y}}-M{\varvec{\delta }}{\mathbf {u}}={}&{\mathbf {f}}-K{\mathbf {y}}^{*}+M{\mathbf {u}}^{*}, \end{aligned}$$

16 $$\begin{aligned} \left( {\mathbf {y}}^{*}-{\mathbf {y}}_{a}\right) \circ {\varvec{\delta }}{\mathbf {z}}_{y,a}+{\mathbf {z}}_{y,a}^{*}\circ {\varvec{\delta }}{\mathbf {y}}={}&\mu {\mathbf {e}}-\left( {\mathbf {y}}^{*}-{\mathbf {y}}_{a}\right) \circ {\mathbf {z}}_{y,a}^{*}, \end{aligned}$$

17 $$\begin{aligned} \left( {\mathbf {y}}_{b}-{\mathbf {y}}^{*}\right) \circ {\varvec{\delta }}{\mathbf {z}}_{y,b}-{\mathbf {z}}_{y,b}^{*}\circ {\varvec{\delta }}{\mathbf {y}}={}&\mu {\mathbf {e}}-\left( {\mathbf {y}}_{b}-{\mathbf {y}}^{*}\right) \circ {\mathbf {z}}_{y,b}^{*}, \end{aligned}$$

18 $$\begin{aligned} \left( {\mathbf {u}}^{*}-{\mathbf {u}}_{a}\right) \circ {\varvec{\delta }}{\mathbf {z}}_{u,a}+{\mathbf {z}}_{u,a}^{*}\circ {\varvec{\delta }}{\mathbf {u}}={}&\mu {\mathbf {e}}-\left( {\mathbf {u}}^{*}-{\mathbf {u}}_{a}\right) \circ {\mathbf {z}}_{u,a}^{*}, \end{aligned}$$

19 $$\begin{aligned} \left( {\mathbf {u}}_{b}-{\mathbf {u}}^{*}\right) \circ {\varvec{\delta }}{\mathbf {z}}_{u,b}-{\mathbf {z}}_{u,b}^{*}\circ {\varvec{\delta }}{\mathbf {u}}={}&\mu {\mathbf {e}}-\left( {\mathbf {u}}_{b}-{\mathbf {u}}^{*}\right) \circ {\mathbf {z}}_{u,b}^{*}. \end{aligned}$$

Here, ${\mathbf {y}}^{*}$, ${\mathbf {u}}^{*}$, ${\varvec{\lambda }}^{*}$, ${\mathbf {z}}_{y,a}^{*}$, ${\mathbf {z}}_{y,b}^{*}$, ${\mathbf {z}}_{u,a}^{*}$, ${\mathbf {z}}_{u,b}^{*}$ denote the most recent Newton iterates for ${\mathbf {y}}$, ${\mathbf {u}}$, ${\varvec{\lambda }}$, ${\mathbf {z}}_{y,a}$, ${\mathbf {z}}_{y,b}$, ${\mathbf {z}}_{u,a}$, ${\mathbf {z}}_{u,b}$, with ${\varvec{\delta }}{\mathbf {y}}$, ${\varvec{\delta }}{\mathbf {u}}$, $\varvec{\delta \lambda }$, ${\varvec{\delta }}{\mathbf {z}}_{y,a}$, ${\varvec{\delta }}{\mathbf {z}}_{y,b}$, ${\varvec{\delta }}{\mathbf {z}}_{u,a}$, ${\varvec{\delta }}{\mathbf {z}}_{u,b}$ the Newton updates, ${\mathbf {e}}$ defines the vector of ones of appropriate dimension, and $\circ$ relates to the multiplication componentwise of two vectors.

In matrix form, (13)–(19) read

$$\begin{aligned}&\left[ \begin{array}{c@{\quad }c@{\quad }c@{\quad }c@{\quad }c@{\quad }c@{\quad }c} M &{} 0 &{} K^{\top } &{} -I &{} I &{} 0 &{} 0 \\ 0 &{} \beta {}M &{} -M &{} 0 &{} 0 &{} -I &{} I \\ K &{} -M &{} 0 &{} 0 &{} 0 &{} 0 &{} 0 \\ Z_{y,a} &{} 0 &{} 0 &{} Y-Y_{a} &{} 0 &{} 0 &{} 0 \\ -Z_{y,b} &{} 0 &{} 0 &{} 0 &{} Y_{b}-Y &{} 0 &{} 0 \\ 0 &{} Z_{u,a} &{} 0 &{} 0 &{} 0 &{} U-U_{a} &{} 0 \\ 0 &{} -Z_{u,b} &{} 0 &{} 0 &{} 0 &{} 0 &{} U_{b}-U \\ \end{array}\right] \left[ \begin{array}{c} {\varvec{\delta }}{\mathbf {y}} \\ {\varvec{\delta }}{\mathbf {u}} \\ \varvec{\delta \lambda } \\ {\varvec{\delta }}{\mathbf {z}}_{y,a} \\ {\varvec{\delta }}{\mathbf {z}}_{y,b} \\ {\varvec{\delta }}{\mathbf {z}}_{u,a} \\ {\varvec{\delta }}{\mathbf {z}}_{u,b} \\ \end{array}\right] \\&\quad =\left[ \begin{array}{c} {\mathbf {y}}_{d}-M{\mathbf {y}}^{*}-K^{\top }{\varvec{\lambda }}^{*}+{\mathbf {z}}_{y,a}^{*}-{\mathbf {z}}_{y,b}^{*} \\ -\beta {}M{\mathbf {u}}^{*}+M{\varvec{\lambda }}^{*}+{\mathbf {z}}_{u,a}^{*}-{\mathbf {z}}_{u,b}^{*} \\ {\mathbf {f}}-K{\mathbf {y}}^{*}+M{\mathbf {u}}^{*} \\ \mu {\mathbf {e}}-({\mathbf {y}}^{*}-{\mathbf {y}}_{a})\circ {\mathbf {z}}_{y,a}^{*} \\ \mu {\mathbf {e}}-({\mathbf {y}}_{b}-{\mathbf {y}}^{*})\circ {\mathbf {z}}_{y,b}^{*} \\ \mu {\mathbf {e}}-({\mathbf {u}}^{*}-{\mathbf {u}}_{a})\circ {\mathbf {z}}_{u,a}^{*} \\ \mu {\mathbf {e}}-({\mathbf {u}}_{b}-{\mathbf {u}}^{*})\circ {\mathbf {z}}_{u,b}^{*} \\ \end{array}\right] , \end{aligned}$$

where *Y*, *U*, $Z_{y,a}$, $Z_{y,b}$, $Z_{u,a}$, $Z_{u,b}$ are diagonal matrices, with the most recent iterates for ${\mathbf {y}}$, ${\mathbf {u}}$, ${\mathbf {z}}_{y,a}$, ${\mathbf {z}}_{y,b}$, ${\mathbf {z}}_{u,a}$, ${\mathbf {z}}_{u,b}$ appearing on the diagonal entries. Similarly, the matrices $Y_{a}$, $Y_{b}$, $U_{a}$, $U_{b}$ are diagonal matrices corresponding to ${\mathbf {y}}_{a}$, ${\mathbf {y}}_{b}$, ${\mathbf {u}}_{a}$, ${\mathbf {u}}_{b}$.

Now, we may write that fourth, fifth, sixth and seventh rows lead to

20 $$\begin{aligned} {\varvec{\delta }}{\mathbf {z}}_{y,a}={}&-(Y-Y_{a})^{-1}Z_{y,a}{\varvec{\delta }}{\mathbf {y}}-{\mathbf {z}}_{y,a}^{*}+\mu (Y-Y_{a})^{-1}{\mathbf {e}}, \end{aligned}$$

21 $$\begin{aligned} {\varvec{\delta }}{\mathbf {z}}_{y,b}={}&(Y_{b}-Y)^{-1}Z_{y,b}{\varvec{\delta }}{\mathbf {y}}-{\mathbf {z}}_{y,b}^{*}+\mu (Y_{b}-Y)^{-1}{\mathbf {e}}, \end{aligned}$$

22 $$\begin{aligned} {\varvec{\delta }}{\mathbf {z}}_{u,a}={}&-(U-U_{a})^{-1}Z_{u,a}{\varvec{\delta }}{\mathbf {u}}-{\mathbf {z}}_{u,a}^{*}+\mu (U-U_{a})^{-1}{\mathbf {e}}, \end{aligned}$$

23 $$\begin{aligned} {\varvec{\delta }}{\mathbf {z}}_{u,b}={}&(U_{b}-U)^{-1}Z_{u,b}{\varvec{\delta }}{\mathbf {u}}-{\mathbf {z}}_{u,b}^{*}+\mu (U_{b}-U)^{-1}{\mathbf {e}}, \end{aligned}$$

whereupon we may consider instead the solution of the reduced system

24 $$\begin{aligned}&\left[ \begin{array}{c@{\quad }c@{\quad }c} M+D_{y} &{} 0 &{} K^{\top } \\ 0 &{} \beta {}M+D_{u} &{} -M \\ K &{} -M &{} 0 \\ \end{array}\right] \left[ \begin{array}{c} {\varvec{\delta }}{\mathbf {y}} \\ {\varvec{\delta }}{\mathbf {u}} \\ \varvec{\delta \lambda } \\ \end{array}\right] \nonumber \\&\quad =\left[ \begin{array}{c} \mu (Y-Y_{a})^{-1}{\mathbf {e}}-\mu (Y_{b}-Y)^{-1}{\mathbf {e}}+{\mathbf {y}}_{d}-M{\mathbf {y}}^{*}-K^{\top }{\varvec{\lambda }}^{*} \\ \mu (U-U_{a})^{-1}{\mathbf {e}}-\mu (U_{b}-U)^{-1}{\mathbf {e}}-\beta {}M{\mathbf {u}}^{*}+M{\varvec{\lambda }}^{*} \\ {\mathbf {f}}-K{\mathbf {y}}^{*}+M{\mathbf {u}}^{*} \\ \end{array}\right] , \end{aligned}$$

where

25 $$\begin{aligned} D_{y}={}&(Y-Y_{a})^{-1}Z_{y,a}+(Y_{b}-Y)^{-1}Z_{y,b}, \end{aligned}$$

26 $$\begin{aligned} D_{u}={}&(U-U_{a})^{-1}Z_{u,a}+(U_{b}-U)^{-1}Z_{u,b}. \end{aligned}$$

The conditions written in (24) are applied, alongside the imposition of (20)–(23), at each Newton iteration.

Note that, due to the fact that state and control bounds are enforced as strict inequalities at each Newton step, the diagonal matrices $D_{y}$ and $D_{u}$ are positive definite.

Of course, it is perfectly natural to consider a problem with only state constraints or only control constraints (or indeed only lower or upper bound constraints). For such cases we may follow exactly the same working to obtain a matrix system of the form (24), removing individual matrices corresponding to constraints that we do not apply.

### Algorithm

We now present the structure of the interior point algorithm, adapted from the paper [17], that we apply to the problems considered in this paper. The essence of the method is to traverse the interior of the feasible region where solutions may arise—we do this by applying a relaxed Newton iteration, reducing the barrier parameter by a factor $\sigma$ at each Newton step. Having computed the Newton updates ${\varvec{\delta }}{\mathbf {y}}$, ${\varvec{\delta }}{\mathbf {u}}$, $\varvec{\delta \lambda }$, ${\varvec{\delta }}{\mathbf {z}}_{y,a}$, ${\varvec{\delta }}{\mathbf {z}}_{y,b}$, ${\varvec{\delta }}{\mathbf {z}}_{u,a}$, ${\varvec{\delta }}{\mathbf {z}}_{u,b}$, we make a step in this direction that also guarantees that the strict bounds are enforced at each iteration. Upon convergence the iterates approach the true solution of the optimization problem, with the additional state and control constraints automatically satisfied.

Let us now consider appropriate stopping criteria for the method. Two natural requirements are for the norms of the primal and dual infeasibilities (at the *k*-th iteration)

$$\begin{aligned} \varvec{\xi }_{p}^{k}={\mathbf {f}}-K{\mathbf {y}}^{k}+M{\mathbf {u}}^{k}, \quad \quad \varvec{\xi }_{d}^{k}=\left[ \begin{array}{c} {\mathbf {y}}_{d}-M{\mathbf {y}}^{k}-K^{\top }{\varvec{\lambda }}^{k} +{\mathbf {z}}_{y,a}^{k}-{\mathbf {z}}_{y,b}^{k} \\ -\beta M{\mathbf {u}}^{k}+M{\varvec{\lambda }}^{k} +{\mathbf {z}}_{u,a}^{k}-{\mathbf {z}}_{u,b}^{k} \end{array}\right] , \end{aligned}$$

to be lower than some prescribed tolerances $\epsilon _{p}$, $\epsilon _{d}$, respectively. Additionally, we require the error in the complementarity products

27 $$\begin{aligned} \varvec{\xi }_{c}^{k} = \left[ \begin{array}{c} \mu {\mathbf {e}}-\left( {\mathbf {y}}^{k}-{\mathbf {y}}_{a}\right) \circ {\mathbf {z}}_{y,a}^{k} \\ \mu {\mathbf {e}}-\left( {\mathbf {y}}_{b}-{\mathbf {y}}^{k}\right) \circ {\mathbf {z}}_{y,b}^{k} \\ \mu {\mathbf {e}}-\left( {\mathbf {u}}^{k}-{\mathbf {u}}_{a}\right) \circ {\mathbf {z}}_{u,a}^{k} \\ \mu {\mathbf {e}}-\left( {\mathbf {u}}_{b}-{\mathbf {u}}^{k}\right) \circ {\mathbf {z}}_{u,b}^{k} \end{array}\right] , \end{aligned}$$

to fall below some specified tolerance $\epsilon _{c}$, and $\mu \le \epsilon _{\mu }$.

We present the algorithm that we apply—its structure is similar to the algorithm outlined in [17, Section 2].
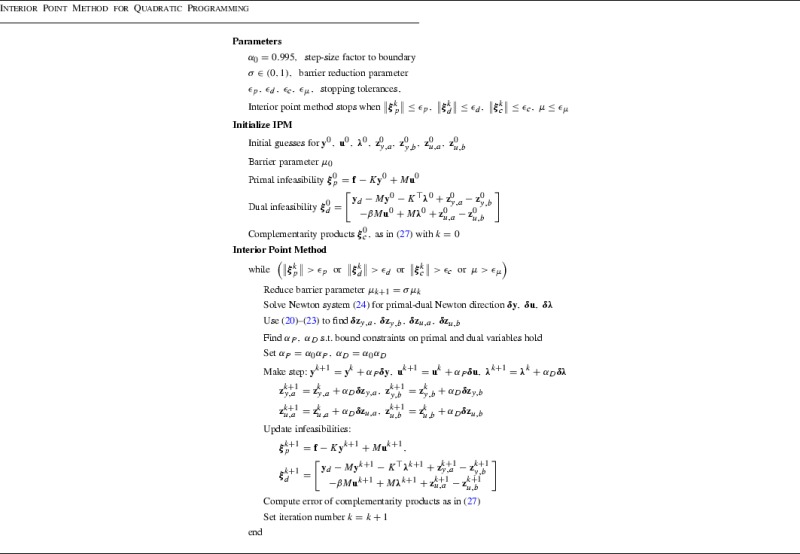


It is clear from the presentation of this method that the dominant computational work arises from the solution of the Newton system (24). It is therefore crucial to construct fast and robust solvers for this system, and this is what we focus on in Sect. 4.

### Time-dependent problems

It is also important to be able to handle time-dependent problems using this methodology, due to the complexity and practical utility of such setups. To provide a brief illustration of how this may be accomplished, let us consider the time-dependent problem:The state, control and adjoint variables are now solved in a space-time domain $\varOmega \times (0,T]$, with ${\mathcal {L}}$ the time-independent component of the PDE operator.

As in [35, 44] for heat equation control problems, we may apply a discretize-then-optimize approach, using the trapezoidal rule to approximate the integrals within the cost functional, and the backward Euler method to account for the time derivative. We thus rewrite the problem in the discrete setting as follows:Here the matrix ${\mathcal {M}}_{1/2}=\text {blkdiag}(\frac{1}{2}M,M,\ldots ,M,\frac{1}{2}M)$, ${\mathcal {M}}=\text {blkdiag}(M,\ldots ,M)$, and

$$\begin{aligned}&{\mathcal {K}}=\left[ \begin{array}{c@{\quad }c@{\quad }c@{\quad }c@{\quad }c} M+\tau {}K &{} &{} &{} &{} \\ -M &{} M+\tau {}K &{} &{} &{} \\ &{} \ddots &{} \ddots &{} &{} \\ &{} &{} -M &{} M+\tau {}K &{} \\ &{} &{} &{} -M &{} M+\tau {}K \\ \end{array}\right] ,\quad \quad \\&\quad {\mathbf {y}}_{d,T}=\left[ \begin{array}{c} \frac{1}{2}{\mathbf {y}}_{d,1} \\ {\mathbf {y}}_{d,2} \\ \vdots \\ {\mathbf {y}}_{d,N_{t}-1} \\ \frac{1}{2}{\mathbf {y}}_{d,N_{t}} \\ \end{array}\right] ,\quad \quad {\mathbf {f}}_{T}=\left[ \begin{array}{c} M{\mathbf {y}}_{0}+{\mathbf {f}} \\ {\mathbf {f}} \\ \vdots \\ {\mathbf {f}} \\ {\mathbf {f}} \\ \end{array}\right] , \end{aligned}$$

where *K* corresponds to the time-independent part of the PDE operator, and $\tau$ denotes the (constant) time-step taken. The vectors ${\mathbf {y}}_{d,i}$ relate to the values of ${\widehat{y}}$ at the *i*-th time-step, and ${\mathbf {y}}_{0}$ is the vector representation of $y_{0}$. We denote by $N_{t}:=\frac{T}{\tau }$ the number of time-steps taken.

We apply Newton iteration to the discrete optimality conditions, in an analogous way to the time-independent problem. This yields the matrix system

28 $$\begin{aligned}&\left[ \begin{array}{c@{\quad }c@{\quad }c@{\quad }c@{\quad }c@{\quad }c@{\quad }c} \tau {\mathcal {M}}_{1/2} &{} 0 &{} {\mathcal {K}}^{\top } &{} -I &{} I &{} 0 &{} 0 \\ 0 &{} \beta \tau {\mathcal {M}}_{1/2} &{} -\tau {\mathcal {M}} &{} 0 &{} 0 &{} -I &{} I \\ {\mathcal {K}} &{} -\tau {\mathcal {M}} &{} 0 &{} 0 &{} 0 &{} 0 &{} 0 \\ Z_{y,a} &{} 0 &{} 0 &{} Y-Y_{a} &{} 0 &{} 0 &{} 0 \\ -Z_{y,b} &{} 0 &{} 0 &{} 0 &{} Y_{b}-Y &{} 0 &{} 0 \\ 0 &{} Z_{u,a} &{} 0 &{} 0 &{} 0 &{} U-U_{a} &{} 0 \\ 0 &{} -Z_{u,b} &{} 0 &{} 0 &{} 0 &{} 0 &{} U_{b}-U \\ \end{array}\right] \quad \left[ \begin{array}{c} {\varvec{\delta }}{\mathbf {y}} \\ {\varvec{\delta }}{\mathbf {u}} \\ \varvec{\delta \lambda } \\ {\varvec{\delta }}{\mathbf {z}}_{y,a} \\ {\varvec{\delta }}{\mathbf {z}}_{y,b} \\ {\varvec{\delta }}{\mathbf {z}}_{u,a} \\ {\varvec{\delta }}{\mathbf {z}}_{u,b} \\ \end{array}\right] \quad \quad \quad \nonumber \\&\quad =\left[ \begin{array}{c} \tau {\mathbf {y}}_{d,T}-\tau {\mathcal {M}}_{1/2}{\mathbf {y}}^{*}-{\mathcal {K}}^{\top }{\varvec{\lambda }}^{*}+{\mathbf {z}}_{y,a}^{*}-{\mathbf {z}}_{y,b}^{*} \\ -\beta \tau {\mathcal {M}}_{1/2}{\mathbf {u}}^{*}+\tau {\mathcal {M}}{\varvec{\lambda }}^{*}+{\mathbf {z}}_{u,a}^{*}-{\mathbf {z}}_{u,b}^{*} \\ {\mathbf {f}}_{T}-{\mathcal {K}}{\mathbf {y}}^{*}+\tau {\mathcal {M}}{\mathbf {u}}^{*} \\ \mu {\mathbf {e}}-\left( {\mathbf {y}}^{*}-{\mathbf {y}}_{a}\right) \circ {\mathbf {z}}_{y,a}^{*} \\ \mu {\mathbf {e}}-\left( {\mathbf {y}}_{b}-{\mathbf {y}}^{*}\right) \circ {\mathbf {z}}_{y,b}^{*} \\ \mu {\mathbf {e}}-\left( {\mathbf {u}}^{*}-{\mathbf {u}}_{a}\right) \circ {\mathbf {z}}_{u,a}^{*} \\ \mu {\mathbf {e}}-\left( {\mathbf {u}}_{b}-{\mathbf {u}}^{*}\right) \circ {\mathbf {z}}_{u,b}^{*} \end{array}\right] , \end{aligned}$$

with $\mathbf {z}_{y,a}$, $\mathbf {z}_{y,b}$, $\mathbf {z}_{u,a}$, $\mathbf {z}_{u,b}$ the same as for the time-independent setting, except now measured over all points in space and time.

Reducing (28) as for the time-independent case gives a block matrix system

29 $$\begin{aligned}&\left[ \begin{array}{c@{\quad }c@{\quad }c} \tau {\mathcal {M}}_{1/2}+{\mathcal {D}}_{y} &{} 0 &{} {\mathcal {K}}^{\top } \\ 0 &{} \beta \tau {\mathcal {M}}_{1/2}+{\mathcal {D}}_{u} &{} -\tau {\mathcal {M}} \\ {\mathcal {K}} &{} -\tau {\mathcal {M}} &{} 0 \\ \end{array}\right] \left[ \begin{array}{c} {\varvec{\delta }}{\mathbf {y}} \\ {\varvec{\delta }}{\mathbf {u}} \\ \varvec{\delta \lambda } \\ \end{array}\right] \nonumber \\&\quad =\left[ \begin{array}{c} \mu (Y-Y_{a})^{-1}{\mathbf {e}}-\mu (Y_{b}-Y)^{-1}{\mathbf {e}}+\tau {\mathbf {y}}_{d,T}-\tau {\mathcal {M}}_{1/2}{\mathbf {y}}^{*}-{\mathcal {K}}^{\top }{\varvec{\lambda }}^{*} \\ \mu (U-U_{a})^{-1}{\mathbf {e}}-\mu (U_{b}-U)^{-1}{\mathbf {e}}-\beta \tau {\mathcal {M}}_{1/2}{\mathbf {u}}^{*}+\tau {\mathcal {M}}{\varvec{\lambda }}^{*} \\ {\mathbf {f}}_{T}-{\mathcal {K}}{\mathbf {y}}^{*}+\tau {\mathcal {M}}{\mathbf {u}}^{*} \end{array}\right] , \end{aligned}$$

with ${\mathcal {D}}_{y}$, ${\mathcal {D}}_{u}$ analogous to $D_{y}$, $D_{u}$, as defined in (25), (26), except with the quantities measured within the entire space-time domain.

## Preconditioning for the Newton system

For the matrix systems considered in this paper, particularly those arising from time-dependent problems, great care must be taken when seeking an appropriate scheme for obtaining an accurate solution. The dimensions of these systems mean that a direct method is often infeasible, so we find that the natural approach is to develop preconditioned Krylov subspace solvers.

When seeking preconditioners for such methods, we exploit the fact that the matrix systems for the PDE-constrained optimization problems are of *saddle point form*:

30 $$\begin{aligned} \underbrace{\left[ \begin{array}{cc} \varPhi &{} \varPsi ^{\top } \\ \varPsi &{} \varTheta \\ \end{array}\right] }_{\mathcal {A}}\left[ \begin{array}{c} {\mathbf {x}}_{1} \\ {\mathbf {x}}_{2} \\ \end{array}\right] =\left[ \begin{array}{c} \mathbf {b}_{1} \\ \mathbf {b}_{2} \\ \end{array}\right] . \end{aligned}$$

Here $\varPhi \in {\mathbb {R}}^{n\times {}n}$, $\varPsi \in {\mathbb {R}}^{m\times {}n}$ and $\varTheta \in {\mathbb {R}}^{m\times {}m}$ (with $m\le {}n$, as in Sect. 2). Further $\varPhi$ and $\varTheta$ are symmetric matrices, meaning that $\mathcal {A}$ is itself symmetric, and all of the matrices are sparse for the finite element method used. We recommend [2] for a thorough overview of saddle point systems and their numerical properties.

The study of preconditioners for systems of this form is a well-established subject area: indeed it is known that two ‘ideal’ preconditioners are given by

$$\begin{aligned} {\mathcal {P}}_{D}=\left[ \begin{array}{c@{\quad }c} \varPhi &{} 0 \\ 0 &{} S \\ \end{array}\right] ,\quad \quad {\mathcal {P}}_{T}=\left[ \begin{array}{c@{\quad }c} \varPhi &{} 0 \\ \varPsi &{} -S \\ \end{array}\right] , \end{aligned}$$

where $S:=-\varTheta +\varPsi \varPhi ^{-1}\varPsi ^{T}$ defines the (negative) *Schur complement* of $\mathcal {A}$. It can be shown [23, 26, 29] that the eigenvalues of the preconditioned systems are given byprovided that these systems are invertible.

In practice, of course, one would not wish to invert $\varPhi$ and *S* exactly within a preconditioner, so the main challenge is to devise effective approximations ${\widehat{\varPhi }}$ and ${\widehat{S}}$ which can be applied within a block diagonal or block triangular preconditioner of the form

31 $$\begin{aligned} {\mathcal {P}}=\left[ \begin{array}{c@{\quad }c} {\widehat{\varPhi }} &{} 0 \\ 0 &{} {\widehat{S}} \\ \end{array}\right] \quad \text {or}\quad \left[ \begin{array}{c@{\quad }c} {\widehat{\varPhi }} &{} 0 \\ \varPsi &{} -{\widehat{S}} \\ \end{array}\right] . \end{aligned}$$

Such preconditioners are very often found to be extremely potent in practice, and in many cases one can prove their effectiveness as well (we discuss this further in Sect. 4.1).

A major objective within the remainder of this paper is to develop effective representations of the (1, 1)-block $\varPhi$ and Schur complement *S* for matrix systems arising from interior point solvers.

### Time-independent problems

We now wish to apply saddle point theory to matrix systems arising from time-independent problems. So consider the matrix system (24), for instance in the case where the matrix *K* arises from a Laplacian operator (considered for Poisson control) or convection-diffusion operator. This system is of saddle point form (30), with

$$\begin{aligned}\varPhi =\left[ \begin{array}{c@{\quad }c} M+D_{y} &{} 0 \\ 0 &{} \beta {}M+D_{u} \\ \end{array}\right] ,\quad \quad \varPsi =\left[ \begin{array}{c@{\quad }c} K &{} -M \\ \end{array}\right] ,\quad \quad \varTheta =\left[ \begin{array}{c} 0 \\ \end{array}\right] . \end{aligned}$$

Let us consider approximating the (1, 1)-block and Schur complement of this matrix system. For this problem *M* is a positive definite matrix, with positive diagonal entries, and the same applies to *K* in the case of Poisson control problems.

We now highlight that mass matrices may in fact be well approximated by their diagonal: for instance, in the case of *Q*1 mass matrices on a uniform two dimensional domain, the eigenvalues of $[\text {diag}(M)]^{-1}M$ are all contained within the interval $[\frac{1}{4},\frac{9}{4}]$ (see [47]). As $D_{y}$ and $D_{u}$ are diagonal and positive definite, one option for approximating $\varPhi$ is hence to take

$$\begin{aligned} {\widehat{\varPhi }}=\left[ \begin{array}{c@{\quad }c} \text {diag}\left( M+D_{y}\right) &{} 0 \\ 0 &{} \text {diag}\left( \beta {}M+D_{u}\right) \\ \end{array}\right] . \end{aligned}$$

The effectiveness of the approximation may be measured in some sense by the eigenvalues of ${\widehat{\varPhi }}^{-1}\varPhi$, which may themselves be determined by the Rayleigh quotient

32 $$\begin{aligned} \frac{{\mathbf {v}}^{\top }\varPhi {\mathbf {v}}}{{\mathbf {v}}^{\top }{\widehat{\varPhi }}{\mathbf {v}}}={}&\frac{{\mathbf {v}}_{1}^{\top }(M+D_{y}){\mathbf {v}}_{1}+{\mathbf {v}}_{2}^{\top }(\beta {}M+D_{u}){\mathbf {v}}_{2}}{{\mathbf {v}}_{1}^{\top }\big [\text {diag}(M+D_{y})\big ]{\mathbf {v}}_{1}+{\mathbf {v}}_{2}^{\top }\big [\text {diag}(\beta {}M+D_{u})\big ]{\mathbf {v}}_{2}} \nonumber \\ ={}&\frac{{\mathbf {v}}_{1}^{\top }M{\mathbf {v}}_{1}+\beta {\mathbf {v}}_{2}^{\top }M{\mathbf {v}}_{2}+{\mathbf {v}}_{1}^{\top }D_{y}{\mathbf {v}}_{1}+{\mathbf {v}}_{2}^{\top }D_{u}{\mathbf {v}}_{2}}{{\mathbf {v}}_{1}^{\top }\big [\text {diag}(M)\big ]{\mathbf {v}}_{1}+\beta {\mathbf {v}}_{2}^{\top }\big [\text {diag}(M)\big ]{\mathbf {v}}_{2}+{\mathbf {v}}_{1}^{\top }D_{y}{\mathbf {v}}_{1}+{\mathbf {v}}_{2}^{\top }D_{u}{\mathbf {v}}_{2}} \nonumber \\ \in {}&\left[ \min \left\{ \frac{{\mathbf {v}}_{1}^{\top }M{\mathbf {v}}_{1}+\beta {\mathbf {v}}_{2}^{\top }M{\mathbf {v}}_{2}}{{\mathbf {v}}_{1}^{\top }\big [\text {diag}(M)\big ]{\mathbf {v}}_{1}+\beta {\mathbf {v}}_{2}^{\top }\big [\text {diag}(M)\big ]{\mathbf {v}}_{2}},1\right\} ,\right. \\&\quad \quad \quad \quad \left. \max \left\{ \frac{{\mathbf {v}}_{1}^{\top }M{\mathbf {v}}_{1}+\beta {\mathbf {v}}_{2}^{\top }M{\mathbf {v}}_{2}}{{\mathbf {v}}_{1}^{\top }\big [\text {diag}(M)\big ]{\mathbf {v}}_{1}+\beta {\mathbf {v}}_{2}^{\top }\big [\text {diag}(M)\big ]{\mathbf {v}}_{2}},1\right\} \right] \nonumber \\ \subseteq&\left[ \min \left\{ \lambda _{\min }\left( \big [\text {diag}(M)\big ]^{-1}M\right) ,1\right\} ,\max \left\{ \lambda _{\max }\left( \big [\text {diag}(M)\big ]^{-1}M\right) ,1\right\} \right] ,\nonumber \end{aligned}$$

where (32) follows from the fact that ${\mathbf {v}}_{1}^{\top }D_{y}{\mathbf {v}}_{1}+{\mathbf {v}}_{2}^{\top }D_{u}{\mathbf {v}}_{2}$ is non-negative. Here ${\mathbf {v}}=\left[ {\mathbf {v}}_{1}^{\top },~{\mathbf {v}}_{2}^{\top }\right] ^{\top }\ne \mathbf {0}$, with ${\mathbf {v}}_{1}$, ${\mathbf {v}}_{2}$ vectors of appropriate length, and $\lambda _{\min }$, $\lambda _{\max }$ denote the smallest and largest eigenvalues of a matrix. We therefore see that if $[\text {diag}(M)]^{-1}M$ is well-conditioned, then the same is true of ${\widehat{\varPhi }}^{-1}\varPhi$.

As an alternative for our approximation ${\widehat{\varPhi }}$, one may apply a Chebyshev semi-iteration method [14, 15, 48] to approximate the inverses of $M+D_{y}$ and $\beta {}M+D_{u}$. This is a slightly more expensive process to approximate this component of the entire system (in general the matrices with the most complex structure are *K* and $K^{\top }$), however due to the tight clustering of the eigenvalues of $[\text {diag}(\varPhi )]^{-1}\varPhi$ we find greater accuracy in the results obtained.

The main task at this stage is to approximate the Schur complement

33 $$\begin{aligned} S=K(M+D_{y})^{-1}K^{\top }+M(\beta {}M+D_{u})^{-1}M. \end{aligned}$$

The aim is to build an approximation such that the eigenvalues of the preconditioned Schur complement are tightly clustered. We motivate our approximation based on a ‘matching’ strategy originally derived in [37] for the Poisson control problem without bound constraints: for this particular problem, *K* is the finite element stiffness matrix, and the matrices $D_{y}=D_{u}=0$. It was shown that by ‘capturing’ both terms ($KM^{-1}K$ and $\frac{1}{\beta }M$) of the Schur complement, one obtains the result

34 $$\begin{aligned} \lambda \left( \left[ \left( K+\frac{1}{\sqrt{\beta }}M \right) M^{-1}\left( K+\frac{1}{\sqrt{\beta }}M\right) \right] ^{-1} \left[ KM^{-1}K+\frac{1}{\beta }M\right] \right) \in \left[ \frac{1}{2},1\right] ,\nonumber \\ \end{aligned}$$

independently of problem size, as well as the value of $\beta$.

Furthermore, it is possible to prove a lower bound of the preconditioned Schur complement for a very general matrix form, as demonstrated below.

#### Theorem 1

Let $S_{G}$ and ${\widehat{S}}_{G}$ be the general matrices

$$\begin{aligned} S_{G}={\bar{X}}{\bar{X}}^{\top }+{\bar{Y}}{\bar{Y}}^{\top },\quad \quad {\widehat{S}}_{G}=({\bar{X}}+{\bar{Y}})({\bar{X}}+{\bar{Y}})^{\top }, \end{aligned}$$

which we assume to be invertible, and with real ${\bar{X}}$, ${\bar{Y}}$. Then the eigenvalues of ${\widehat{S}}_{G}^{-1}S_{G}$ are real, and satisfy $\lambda \ge \frac{1}{2}$.

#### Proof

As $S_{G}$ and ${\widehat{S}}_{G}$ are invertible, they are symmetric positive definite by constuction. To examine the spectrum of ${\widehat{S}}_{G}^{-1}S_{G}$ we therefore consider the Rayleigh quotient (for real ${\mathbf {v}}\ne \mathbf {0}$):

$$\begin{aligned} \ R:=\frac{{\mathbf {v}}^{\top }S_{G}{\mathbf {v}}}{{\mathbf {v}}^{\top } {\widehat{S}}_{G}{\mathbf {v}}}=\frac{{\varvec{\chi }}^{\top }{\varvec{\chi }} +{\varvec{\omega }}^{\top }{\varvec{\omega }}}{({\varvec{\chi }} +{\varvec{\omega }})^{\top }({\varvec{\chi }} +{\varvec{\omega }})},\quad \quad \quad {\varvec{\chi }} ={\bar{X}}^{\top }{\mathbf {v}},\quad {\varvec{\omega }}={\bar{Y}}^{\top }{\mathbf {v}}, \end{aligned}$$

which is itself clearly real. By the invertibility of $S_{G}$ and ${\widehat{S}}_{G}$, both numerator and denominator are positive. Therefore

$$\begin{aligned} \frac{1}{2}({\varvec{\chi }}-{\varvec{\omega }})^{\top }({\varvec{\chi }} -{\varvec{\omega }})\ge 0\quad \Leftrightarrow \quad {\varvec{\chi }}^{\top } {\varvec{\chi }}+{\varvec{\omega }}^{\top }{\varvec{\omega }}\ge \frac{1}{2} ({\varvec{\chi }}+{\varvec{\omega }})^{\top }({\varvec{\chi }} +{\varvec{\omega }})\quad \Leftrightarrow \quad {}R\ge \frac{1}{2}, \end{aligned}$$

which gives the result. $\square$

For the Schur complement given by (33), the matrices ${\bar{X}}=K(M+D_{y})^{-1/2}$ and ${\bar{Y}}=M(\beta {}M+D_{u})^{-1/2}$, which we use below to derive our approximation. Note that to demonstrate an upper bound for this problem, one would write

35 $$\begin{aligned} R={}&1-\frac{2{\varvec{\omega }}^{\top }{\varvec{\chi }}}{({\varvec{\chi }}+{\varvec{\omega }})^{\top }({\varvec{\chi }}+{\varvec{\omega }})} \nonumber \\ ={}&1-\frac{2{\mathbf {v}}^{\top }M(\beta {}M+D_{u})^{-1/2}(M+D_{y})^{-1/2}K^{\top }{\mathbf {v}}}{{\mathbf {v}}^{\top }K(M+D_{y})^{-1}K^{\top }{\mathbf {v}}+{\mathbf {v}}^{\top }M(\beta {}M+D_{u})^{-1}M{\mathbf {v}}+2{\mathbf {v}}^{\top }M(\beta {}M+D_{u})^{-1/2}(M+D_{y})^{-1/2}K^{\top }{\mathbf {v}}} \nonumber \\ \le {}&1-\min _{{\mathbf {v}}\ne \mathbf {0}}\left\{ \frac{2{\mathbf {v}}^{\top }M(\beta {}M+D_{u})^{-1/2}(M+D_{y})^{-1/2}K^{\top }{\mathbf {v}}}{{\mathbf {v}}^{\top }\left[ K(M+D_{y})^{-1}K^{\top }+M(\beta {}M+D_{u})^{-1}M+2M(\beta {}M+D_{u})^{-1/2}(M+D_{y})^{-1/2}K^{\top }\right] {\mathbf {v}}}\right\} \nonumber \\ ={}&1-\min _{{\mathbf {v}}\ne \mathbf {0}}\left\{ \left( 1+\frac{{\mathbf {v}}^{\top }\left[ K(M+D_{y})^{-1}K^{\top }+M(\beta {}M+D_{u})^{-1}M\right] {\mathbf {v}}}{2{\mathbf {v}}^{\top }M(\beta {}M+D_{u})^{-1/2}(M+D_{y})^{-1/2}K^{\top }{\mathbf {v}}}\right) ^{-1}\right\} , \end{aligned}$$

provided ${\mathbf {v}}\notin \text {ker}(K^{\top })$. We may therefore draw the following conclusions:

- The Rayleigh quotient *R* is certainly finite, as the case ${\varvec{\chi }}+{\varvec{\omega }}=\mathbf {0}$ is disallowed by the assumption of invertibility of ${\widehat{S}}_{G}$.
- Furthermore, depending on the (typically unknown) entries of $D_{y}$, the term ${\mathbf {v}}^{\top }K(M+D_{y})^{-1}K^{\top }{\mathbf {v}}$ should be large compared with the term ${\mathbf {v}}^{\top }M(\beta {}M+D_{u})^{-1/2}(M+D_{y})^{-1/2}K^{\top }{\mathbf {v}}$ arising in the denominator above, due to the fact that *K* has larger eigenvalues than *M* in general. The term being minimized in (35) will therefore not take a large negative value in general, and hence *R* will not become excessively large.
- However, it is generally not possible to demonstrate a concrete upper bound unless ${\bar{X}}$ and ${\bar{Y}}$ have structures which can be exploited. The reason for this is that the diagonal matrices $D_{y}$ and $D_{u}$ that determine the distribution of the eigenvalues can take any positive value (including arbitrarily small or infinitely large values, in finite precision), depending on the behaviour of the Newton iterates, which is impossible to control. In practice, we find it is rare for the largest eigenvalues of the preconditioned Schur complement to exceed values of roughly $5-10$.
- However, using the methodology of Theorem 1, results of this form have been demonstrated for problems such as convection-diffusion control [36] and heat equation control [35] (without additional bound constraints). We also highlight that, in [39, 42], preconditioners for problems with bound constraints^1^, solved with active set Newton methods, are derived. In [39], parameter-independent bounds are derived for a preconditioned Schur complement, however the additional requirement is imposed that *M* is a *lumped* (i.e. diagonal) mass matrix. As we do not assume that the mass matrices are lumped in this work, we may not exploit this method to obtain an upper eigenvalue bound.
- In general, the eigenvalues of ${\widehat{S}}_{G}^{-1}S_{G}$ are better clustered if the term ${\bar{X}}{\bar{Y}}^{\top }+{\bar{Y}}{\bar{X}}^{\top }$ is positive semi-definite, or ‘nearly’ positive semi-definite. The worst case would arise in the setting where ${\varvec{\chi }}\approx -{\varvec{\omega }}$, however for our problem the matrices ${\bar{X}}$ and ${\bar{Y}}$ do not relate closely to each other as the activities in the state and control variables do not share many common features.

We now provide an indicative result for the situation which corresponds to the limiting case when the barrier parameter $\mu \rightarrow 0$ and all state and control bounds are satisfied as strict inequalities, i.e. all bounds remain inactive at the optimum. In such a case all Lagrange multipliers ${\mathbf {z}}_{y,a}$, ${\mathbf {z}}_{y,b}$, ${\mathbf {z}}_{u,a}$ and ${\mathbf {z}}_{u,b}$ would take small values of order $\mu$ and so would the diagonal matrices $D_{y}$ and $D_{u}$ defined by (25) and (26), respectively. In the limit we would observe $D_{y}=0$ and $D_{u}=0$.

#### Lemma 1

If $D_{y}=D_{u}=0$, and the matrix $K+K^{\top }$ is positive semi-definite^2^, then the eigenvalues of ${\widehat{S}}_{G}^{-1}S_{G}$ satisfy $\lambda \le 1$.

#### Proof

From the above working, we have that

$$\begin{aligned} R=1-\frac{2{\varvec{\omega }}^{\top }{\varvec{\chi }}}{({\varvec{\chi }} +{\varvec{\omega }})^{\top }({\varvec{\chi }}+{\varvec{\omega }})}=1 -\frac{\frac{1}{\sqrt{\beta }}{\mathbf {v}}^{\top }(K+K^{\top }){\mathbf {v}}}{{\mathbf {v}}^{\top }KM^{-1}K^{\top }{\mathbf {v}}+\frac{1}{\beta } {\mathbf {v}}^{\top }M{\mathbf {v}}+\frac{1}{\sqrt{\beta }}{\mathbf {v}}^{\top }(K +K^{\top }){\mathbf {v}}}, \end{aligned}$$

using the assumption that $D_{y}=D_{u}=0$. The denominator of the quotient above is clearly positive, due to the positive definiteness of *M*, and the numerator is non-negative by the assumption of positive semi-definiteness of $K+K^{\top }$. This automatically leads to the statement $R\le 1$, and hence the result. $\square$

The ‘matching strategy’ presented here guarantees a lower bound for the preconditioned Schur complement of matrices of this form, provided some very weak assumptions hold^3^, and often results in the largest eigenvalue being of moderate magnitude. We therefore wish to make use of this matching approach to generate effective Schur complement approximations for the very general class of matrix systems considered in this manuscript. In particular, we consider matrices *K* of general form (as opposed to the stiffness matrix as in (34)), as well as diagonal matrices $D_{y}$ and $D_{u}$ which can be extremely ill-conditioned. Motivated by Theorem 1, we may therefore consider a matching strategy for the Schur complement (33), by writing

36 $$\begin{aligned} {\widehat{S}}_{1}:=\big (K+{\widehat{M}}_{1}\big )(M+D_{y})^{-1}\big (K +{\widehat{M}}_{1}\big )^{\top }, \end{aligned}$$

where ${\widehat{M}}_{1}$ is chosen such that the matrix  captures the second term of the exact Schur complement (33). That is,This leads to the following requirement when selecting ${\widehat{M}}_{1}$:

$$\begin{aligned} {\widehat{M}}_{1}\approx {}M(\beta {}M+D_{u})^{-1/2}(M+D_{y})^{1/2}. \end{aligned}$$

We take diagonal approximations where appropriate, in order to avoid having to construct square roots of matrices, which would be extremely expensive computationally. That is, we take

37 $$\begin{aligned} {\widehat{M}}_{1}=M\big [\text {diag}(\beta {}M+D_{u})\big ]^{-1/2} \big [\text {diag}(M+D_{y})\big ]^{1/2}. \end{aligned}$$

We now present a result concerning this choice for ${\widehat{M}}_{1}$.

#### Lemma 2

When the Schur complement (33) is approximated by ${\widehat{S}}_{1}$, and with ${\widehat{M}}_{1}$ given by (37), then, provided $K+{\widehat{M}}_{1}$ is invertible, the eigenvalues of ${\widehat{S}}_{1}^{-1}S$ satisfy

$$\begin{aligned} \lambda \ge \frac{1}{2}\cdot \frac{\min \left\{ \lambda _{\min } \left( \big [ diag (M)\big ]^{-1}M\right) ,1\right\} }{\max \left\{ \lambda _{\max }\left( \big [ diag (M)\big ]^{-1}M\right) ,1\right\} }. \end{aligned}$$

In other words the eigenvalues are bounded below by a fixed constant, depending only on the finite element discretization used.

#### Proof

Selecting ${\widehat{M}}_{1}$ as in (37) gives that the eigenvalues of ${\widehat{S}}_{1}^{-1}S$ are determined by the Rayleigh quotientwhere for this problem the vectors of interest are ${\varvec{\chi }}=(M+D_{y})^{-1/2}K^{\top }{\mathbf {v}}$, ${\varvec{\omega }}=(\beta {}M+D_{u})^{-1/2}M{\mathbf {v}}$ and $\varvec{\gamma }=(M+D_{y})^{-1/2}\left[ \text {diag}(M+D_{y})\right] ^{1/2} \left[ \text {diag}(\beta {}M+D_{u})\right] ^{-1/2}M{\mathbf {v}}$. As the numerator and denominator both consist of positive quantities, using the assumption that $K+{\widehat{M}}_{1}$ is invertible, with the possible exception of ${\varvec{\chi }}^{\top }{\varvec{\chi }}$ which may be zero, we can state that

$$\begin{aligned} R\ge \min \left\{ \frac{{\varvec{\omega }}^{\top }{\varvec{\omega }}}{\varvec{\gamma }^{\top } \varvec{\gamma }},1\right\} \cdot \frac{{\varvec{\chi }}^{\top } {\varvec{\chi }}+\varvec{\gamma }^{\top }\varvec{\gamma }}{({\varvec{\chi }}+\varvec{\gamma })^{\top }({\varvec{\chi }}+\varvec{\gamma })}\ge \frac{1}{2}\cdot \min \left\{ \frac{{\varvec{\omega }}^{\top }{\varvec{\omega }}}{\varvec{\gamma }^{\top }\varvec{\gamma }},1\right\} , \end{aligned}$$

by setting ${\bar{X}}=K(M+D_{y})^{-1/2}$ and ${\bar{Y}}=M\left[ \text {diag}(\beta {}M+D_{u})\right] ^{-1/2}\left[ \text {diag} (M+D_{y})\right] ^{1/2}(M+D_{y})^{-1/2}$ within Theorem 1.

We then observe that the quotient $\frac{{\varvec{\omega }}^{\top }{\varvec{\omega }}}{\varvec{\gamma }^{\top }\varvec{\gamma }}$ can be decomposed as

$$\begin{aligned}&\frac{{\mathbf {w}}_{1}^{\top }(\beta {}M+D_{u})^{-1}{\mathbf {w}}_{1}}{{\mathbf {w}}_{1}^{\top }\left[ \text {diag}(\beta {}M+D_{u})\right] ^{-1/2}\left[ \text {diag}(M+D_{y})\right] ^{1/2}(M+D_{y})^{-1}\left[ \text {diag}(M+D_{y})\right] ^{1/2}\left[ \text {diag}(\beta {}M+D_{u})\right] ^{-1/2}{\mathbf {w}}_{1}} \\&\quad ={}\frac{{\mathbf {w}}_{1}^{\top }(\beta {}M+D_{u})^{-1}{\mathbf {w}}_{1}}{{\mathbf {w}}_{1}^{\top }\left[ \text {diag}(\beta {}M+D_{u})\right] ^{-1}{\mathbf {w}}_{1}}\cdot \frac{{\mathbf {w}}_{2}^{\top }\left[ \text {diag}(M+D_{y})\right] ^{-1}{\mathbf {w}}_{2}}{{\mathbf {w}}_{2}^{\top }(M+D_{y})^{-1}{\mathbf {w}}_{2}}, \end{aligned}$$

where $${\mathbf {w}}_{1}=M{\mathbf {v}}\ne \mathbf {0}$$ and $${\mathbf {w}}_{2}=\left[ \text {diag}(M+D_{y})\right] ^{1/2}\left[ \text {diag}(\beta {}M+D_{u})\right] ^{-1/2}{\mathbf {w}}_{1}\ne \mathbf {0}$$.

Now, it may be easily shown that

$$\begin{aligned} \frac{{\mathbf {w}}_{1}^{\top }(\beta {}M+D_{u})^{-1}{\mathbf {w}}_{1}}{{\mathbf {w}}_{1}^{\top }\left[ \text {diag}(\beta {}M+D_{u})\right] ^{-1}{\mathbf {w}}_{1}}\ge {}&\left[ \max \left\{ \lambda _{\max }\left( \big [\text {diag}(M)\big ]^{-1}M\right) ,1\right\} \right] ^{-1}, \\ \frac{{\mathbf {w}}_{2}^{\top }\left[ \text {diag}(M+D_{y})\right] ^{-1}{\mathbf {w}}_{2}}{{\mathbf {w}}_{2}^{\top }(M+D_{y})^{-1}{\mathbf {w}}_{2}}\ge {}&\min \left\{ \lambda _{\min }\left( \big [\text {diag}(M)\big ]^{-1}M\right) ,1\right\} , \end{aligned}$$

using the working earlier in this section. Combining these bounds gives the desired result. $\square$

Clearly, it is valuable to have this insight that using our approximation ${\widehat{M}}_{1}$ retains the parameter independence of the lower bound for the eigenvalues of ${\widehat{S}}_{1}^{-1}S$. We note that this can potentially be a weak bound, as the large diagonal entries in $D_{y}$ and $D_{u}$ are likely to dominate the behaviour of $M+D_{y}$ and $\beta {}M+D_{u}$, thus driving the eigenvalues of the preconditioned Schur complement closer to 1.

We highlight that, in practice, one may also approximate the inverses of $K+{\widehat{M}}_{1}$ and its transpose effectively using a multigrid process. We apply the Aggregation-based Algebraic Multigrid (AGMG) software [30–33] for this purpose within our iterative solvers.

Combining our approximations of $\varPhi$ and *S*, we propose the following block diagonal preconditioner of the form (31):

$$\begin{aligned} {\mathcal {P}}_{1}=\left[ \begin{array}{c@{\quad }c@{\quad }c} (M+D_{y})_{\text {approx}} &{} 0 &{} 0 \\ 0 &{} (\beta {}M+D_{u})_{\text {approx}} &{} 0 \\ 0 &{} 0 &{} {\widehat{S}}_{1} \\ \end{array}\right] , \end{aligned}$$

where $(M+D_{y})_{\text {approx}}$, $(\beta {}M+D_{u})_{\text {approx}}$ indicate our choice of approximations for $M+D_{y}$, $\beta {}M+D_{u}$ (i.e. diagonal approximation, or Chebyshev semi-iteration method), and ${\widehat{S}}_{1}$ is given by (36). This preconditioner is symmetric positive definite, and may thus be applied within a symmetric solver such as Minres [34].

It is useful to consider the distribution of eigenvalues of the preconditioned system, as this will control the convergence properties of the Minres method. The fundamental result we use for our analysis of saddle point matrices (30) is stated below [40, Lemma 2.1].

#### Theorem 2

If $\varPhi$ is symmetric positive definite, $\varPsi$ is full rank, and $\varTheta =0$, the eigenvalues of $\mathcal {A}$ are contained within the following intervals:

$$\begin{aligned} \lambda (\mathcal {A})\in {}&\left[ \frac{1}{2}\left( \mu _{\min }-\sqrt{\mu _{\min }^{2}+4\sigma _{\max }^{2}}\right) ,\frac{1}{2}\left( \mu _{\max }-\sqrt{\mu _{\max }^{2}+4\sigma _{\min }^{2}}\right) \right] \\&\quad \cup \left[ \mu _{\min },\frac{1}{2}\left( \mu _{\max }+\sqrt{\mu _{\max }^{2}+4\sigma _{\max }^{2}}\right) \right] , \end{aligned}$$

where $\mu _{\max }$, $\mu _{\min }$ denote the largest and smallest eigenvalues of $\varPhi$, with $\sigma _{\max }$, $\sigma _{\min }$ the largest and smallest singular values of $\varPsi$.

We now wish to apply a result of this form to the preconditioned system. The preconditioned matrix, when a general block diagonal preconditioner of the form (31) is used, is given by

$$\begin{aligned} \ {\mathcal {P}}^{-1}\mathcal {A}=\left[ \begin{array}{c@{\quad }c} {\widehat{\varPhi }} &{} 0 \\ 0 &{} {\widehat{S}} \\ \end{array}\right] ^{-1}\left[ \begin{array}{c@{\quad }c} \varPhi &{} \varPsi ^{\top } \\ \varPsi &{} 0 \\ \end{array}\right] =\left[ \begin{array}{c@{\quad }c} {\widehat{\varPhi }}^{-1}\varPhi &{} {\widehat{\varPhi }}^{-1}\varPsi ^{\top } \\ {\widehat{S}}^{-1}\varPsi &{} 0 \\ \end{array}\right] . \end{aligned}$$

Now, to analyse the properties of this system, let

$$\begin{aligned} \lambda ({\widehat{\varPhi }}^{-1}\varPhi )\in [\phi _{\min },\phi _{\max }], \quad \quad \lambda ({\widehat{S}}^{-1}S)\in [s_{\min },s_{\max }], \end{aligned}$$

where $\phi _{\min },s_{\min }>0$. The analysis of this section gives us information about these values.

By the similarity property of matrix systems (using that for our problem ${\widehat{\varPhi }}$ and ${\widehat{S}}$ are positive definite) the eigenvalues will be the same as those of

$$\begin{aligned} \ {\mathcal {P}}^{-1/2}\mathcal {A}{\mathcal {P}}^{-1/2}={}&\left[ \begin{array}{c@{\quad }c} {\widehat{\varPhi }}^{-1/2} &{} 0 \\ 0 &{} {\widehat{S}}^{-1/2} \\ \end{array}\right] \left[ \begin{array}{c@{\quad }c} \varPhi &{} \varPsi ^{\top } \\ \varPsi &{} 0 \\ \end{array}\right] \left[ \begin{array}{c@{\quad }c} {\widehat{\varPhi }}^{-1/2} &{} 0 \\ 0 &{} {\widehat{S}}^{-1/2} \\ \end{array}\right] \\ \ ={}&\left[ \begin{array}{c@{\quad }c} {\widehat{\varPhi }}^{-1/2}\varPhi {\widehat{\varPhi }}^{-1/2} &{} {\widehat{\varPhi }}^{-1/2}\varPsi ^{\top }{\widehat{S}}^{-1/2} \\ {\widehat{S}}^{-1/2}\varPsi {\widehat{\varPhi }}^{-1/2} &{} 0 \\ \end{array}\right] . \end{aligned}$$

The eigenvalues of the (1, 1)-block of this matrix, ${\widehat{\varPhi }}^{-1/2}\varPhi {\widehat{\varPhi }}^{-1/2}$, are the same as those of ${\widehat{\varPhi }}^{-1}\varPhi$ by similarity, and so are contained in $[\phi _{\min },\phi _{\max }]$. The singular values of the (2, 1)-block are given by the square roots of the eigenvalues of ${\widehat{S}}^{-1/2}\varPsi {\widehat{\varPhi }}^{-1}\varPsi ^{\top }{\widehat{S}}^{-1/2}$, i.e. the square roots of the eigenvalues of ${\widehat{S}}^{-1}(\varPsi {\widehat{\varPhi }}^{-1}\varPsi ^{\top })$ by similarity. Writing the Rayleigh quotient (for ${\mathbf {v}}\ne \mathbf {0}$),

$$\begin{aligned} \frac{{\mathbf {v}}^{\top }\varPsi {\widehat{\varPhi }}^{-1}\varPsi ^{\top } {\mathbf {v}}}{{\mathbf {v}}^{\top }{\widehat{S}}{\mathbf {v}}}=\frac{{\mathbf {v}}^{\top }\varPsi {\widehat{\varPhi }}^{-1}\varPsi ^{\top }{\mathbf {v}}}{{\mathbf {v}}^{\top }\varPsi \varPhi ^{-1}\varPsi ^{\top }{\mathbf {v}}}\cdot \frac{{\mathbf {v}}^{\top }\varPsi \varPhi ^{-1}\varPsi ^{\top }{\mathbf {v}}}{{\mathbf {v}}^{\top }{\widehat{S}}{\mathbf {v}}}=\underbrace{\frac{\bar{{\mathbf {v}}}^{\top }{\widehat{\varPhi }}^{-1}\bar{{\mathbf {v}}}}{\bar{{\mathbf {v}}}^{\top }\varPhi ^{-1}\bar{{\mathbf {v}}}}}_{\in [\phi _{\min },\phi _{\max }]}\cdot \underbrace{\frac{{\mathbf {v}}^{\top }\varPsi \varPhi ^{-1}\varPsi ^{\top }{\mathbf {v}}}{{\mathbf {v}}^{\top }{\widehat{S}}{\mathbf {v}}}}_{\in [s_{\min },s_{\max }]}, \end{aligned}$$

where $\bar{{\mathbf {v}}}=\varPsi ^{\top }{\mathbf {v}}$, enables us to pin the singular values of the (2, 1)-block within .

So, using Theorem 2, the eigenvalues of ${\mathcal {P}}^{-1}\mathcal {A}$ are contained within the interval stated below.

#### Lemma 3

If $\varPhi$ and *S* are symmetric positive definite, and the above bounds on $\lambda ({\widehat{\varPhi }}^{-1}\varPhi )$ and $\lambda ({\widehat{S}}^{-1}S)$ hold, then the eigenvalues of ${\mathcal {P}}^{-1}\mathcal {A}$ satisfy

$$\begin{aligned} \lambda ({\mathcal {P}}^{-1}\mathcal {A})\in & {} \left[ \frac{1}{2}\left( \phi _{\min }-\sqrt{\phi _{\min }^{2}+4\phi _{\max }s_{\max }}\right) ,\frac{1}{2}\left( \phi _{\max }-\sqrt{\phi _{\max }^{2}+4\phi _{\min }s_{\min }}\right) \right] \\&\quad \cup \left[ \phi _{\min },\frac{1}{2}\left( \phi _{\max }+\sqrt{\phi _{\max }^{2}+4\phi _{\max }s_{\max }}\right) \right] . \end{aligned}$$

It is therefore clear that, for our problem, a good approximation of the Schur complement will guarantee clustered eigenvalues of the preconditioned system, and therefore rapid convergence of the Minres method. As we have observed for our problem, the quantities of interest are therefore the largest eigenvalues of ${\widehat{S}}^{-1}S$, which can vary at every step of a Newton method.

We now present a straightforward result concerning the eigenvectors of a preconditioned saddle point system of the form under consideration.

#### Proposition 1

Consider an eigenvalue $\lambda$ that satisfies

38 $$\begin{aligned} \left[ \begin{array}{c@{\quad }c} \varPhi &{} \varPsi ^{\top } \\ \varPsi &{} 0 \\ \end{array}\right] \left[ \begin{array}{c} {\mathbf {v}}_{1} \\ {\mathbf {v}}_{2} \\ \end{array}\right] =\lambda \left[ \begin{array}{c@{\quad }c} {\widehat{\varPhi }} &{} 0 \\ 0 &{} {\widehat{S}} \\ \end{array}\right] \left[ \begin{array}{c} {\mathbf {v}}_{1} \\ {\mathbf {v}}_{2} \\ \end{array}\right] , \end{aligned}$$

with $\varPhi$, $S=\varPsi \varPhi ^{-1}\varPsi ^{\top }$, ${\widehat{\varPhi }}$, ${\widehat{S}}$ symmetric positive definite. Then either $\lambda$ is an eigenvalue of ${\widehat{\varPhi }}^{-1}\varPhi$, or $\lambda$, ${\mathbf {v}}_{1}$ and ${\mathbf {v}}_{2}$ satisfy

$$\begin{aligned} \left( \lambda {\widehat{\varPhi }}-\varPhi -\frac{1}{\lambda }\varPsi ^{\top } {\widehat{S}}^{-1}\varPsi \right) {\mathbf {v}}_{1}=\mathbf {0},\quad \quad {\mathbf {v}}_{2}=\frac{1}{\lambda }{\widehat{S}}^{-1}\varPsi {\mathbf {v}}_{1}. \end{aligned}$$

#### Proof

Equation (38) is equivalent to

39 $$\begin{aligned} \varPsi ^{\top }{\mathbf {v}}_{2}={}&\big (\lambda {\widehat{\varPhi }}-\varPhi \big ){\mathbf {v}}_{1}, \end{aligned}$$

40 $$\begin{aligned} \varPsi {\mathbf {v}}_{1}={}&\lambda {\widehat{S}}{\mathbf {v}}_{2}. \end{aligned}$$

Let us first consider the case where $\varPsi {\mathbf {v}}_{1}=\mathbf {0}$ (there are at most $n-m$ such linearly independent vectors that correspond to eigenvectors). Then (40) tells us that ${\mathbf {v}}_{2}=\mathbf {0}$, from which we conclude from (39) that $(\lambda {\widehat{\varPhi }}-\varPhi ){\mathbf {v}}_{1}=\mathbf {0}$. Therefore, in this case, the eigenvalues are given by eigenvalues of ${\widehat{\varPhi }}^{-1}\varPhi$, with eigenvectors of the form $\left[ {\mathbf {v}}_{1}^{\top },~\mathbf {0}^{\top }\right] ^{\top }$—there are at most $n-m$ such solutions.

If $\varPsi {\mathbf {v}}_{1}\ne \mathbf {0}$, we may rearrange (40) to obtain

$$\begin{aligned} {\mathbf {v}}_{2}=\frac{1}{\lambda }{\widehat{S}}^{-1}\varPsi {\mathbf {v}}_{1}\quad \Rightarrow \quad \varPsi ^{\top }{\mathbf {v}}_{2}=\frac{1}{\lambda }\varPsi ^{\top }{\widehat{S}}^{-1}\varPsi {\mathbf {v}}_{1}, \end{aligned}$$

which we may substitute into (39) to obtain

$$\begin{aligned} \frac{1}{\lambda }\varPsi ^{\top }{\widehat{S}}^{-1}\varPsi {\mathbf {v}}_{1} =\big (\lambda {\widehat{\varPhi }}-\varPhi \big ){\mathbf {v}}_{1}. \end{aligned}$$

This may be trivially rearranged to obtain the required result. $\square$

We observe that the eigenvalues and eigenvectors of the (1, 1)-block and Schur complement (along with their approximations) interact strongly with each other. This decreases the likelihood of many extreme eigenvalues of ${\widehat{S}}^{-1}S$ arising in practice, as this would have implications on the numerical properties of $\varPhi$ and $\varPsi$ (which for our problems do not interact at all strongly). However the working provided here shows that this is very difficult to prove rigorously, due to the wide generality of the saddle point systems being examined—we must also rely on the physical properties of the PDE operators within the optimization framework. Our numerical experiments of Sect. 5 indicate that the eigenvalues of ${\widehat{S}}^{-1}S$, and therefore the preconditioned system, are tightly clustered, matching some of the observations made in this section.

As an alternative to the block diagonal preconditioner ${\mathcal {P}}_{1}$, we may take account of information on the block lower triangular parts of the matrix system, and apply the block triangular preconditioner

$$\begin{aligned} \ {\mathcal {P}}_{2}=\left[ \begin{array}{c@{\quad }c@{\quad }c} (M+D_{y})_{\text {approx}} &{} 0 &{} 0 \\ 0 &{} (\beta {}M+D_{u})_{\text {approx}} &{} 0 \\ K &{} -M &{} -{\widehat{S}}_{1} \\ \end{array}\right] , \end{aligned}$$

within a non-symmetric solver such as Gmres [41].

It is possible to carry out eigenvalue analysis for the block triangular preconditioner ${\mathcal {P}}_{2}$ in the same way as for the block diagonal preconditioner ${\mathcal {P}}_{1}$. However it is well known that the convergence of non-symmetric solvers such as Gmres does not solely depend on the eigenvalues of the preconditioned system, and therefore such an analysis would be less useful in practice.

We now consider a completely different strategy for preconditioning the matrix system. We may first rearrange (24) to the form

41 $$\begin{aligned}&\left[ \begin{array}{c@{\quad }c@{\quad }c} \beta {}M+D_{u} &{} -M &{} 0 \\ -M &{} 0 &{} K \\ 0 &{} K^{\top } &{} M+D_{y} \\ \end{array}\right] \left[ \begin{array}{c} {\varvec{\delta }}{\mathbf {u}} \\ \varvec{\delta \lambda } \\ {\varvec{\delta }}{\mathbf {y}} \\ \end{array}\right] \\&\quad =\left[ \begin{array}{c} \mu (U-U_{a})^{-1}{\mathbf {e}}-\mu (U_{b}-U)^{-1}{\mathbf {e}}-\beta {}M{\mathbf {u}}^{*}+M{\varvec{\lambda }}^{*} \\ {\mathbf {f}}-K{\mathbf {y}}^{*}+M{\mathbf {u}}^{*} \\ \mu (Y-Y_{a})^{-1}{\mathbf {e}}-\mu (Y_{b}-Y)^{-1}{\mathbf {e}}+{\mathbf {y}}_{d}-M{\mathbf {y}}^{*}-K^{\top }{\varvec{\lambda }}^{*} \\ \end{array}\right] .\nonumber \end{aligned}$$

The matrix within (41) is a saddle point system of the form (30), with

$$\begin{aligned} \ \varPhi =\left[ \begin{array}{c@{\quad }c} \beta {}M+D_{u} &{} -M \\ -M &{} 0 \\ \end{array}\right] ,\quad \quad \varPsi =\left[ \begin{array}{c@{\quad }c} 0 &{} K^{\top } \\ \end{array}\right] ,\quad \quad \varTheta =\left[ \begin{array}{c} M+D_{y} \\ \end{array}\right] . \end{aligned}$$

This approach also has the desirable feature that the (1, 1)-block $\varPhi$ can be inverted almost precisely, as all that is required is a method for approximating the inverse of a mass matrix (to be applied twice). Once again, a very cheap and accurate method is Chebyshev semi-iteration [14, 15, 48], so we apply this strategy within our preconditioner.

Once again, the main challenge is to approximate the Schur complement:

$$\begin{aligned} \ S={}&-(M+D_{y})+\left[ \begin{array}{c@{\quad }c} 0 &{} K^{\top } \\ \end{array}\right] \left[ \begin{array}{c@{\quad }c} \beta {}M+D_{u} &{} -M \\ -M &{} 0 \\ \end{array}\right] ^{-1}\left[ \begin{array}{c} 0 \\ K \\ \end{array}\right] \\ \ ={}&-(M+D_{y})+\left[ \begin{array}{c@{\quad }c} 0 &{} K^{\top } \\ \end{array}\right] \left[ \begin{array}{c@{\quad }c} 0 &{} -M^{-1} \\ -M^{-1} &{} -M^{-1}(\beta {}M+D_{u})M^{-1} \\ \end{array}\right] \left[ \begin{array}{c} 0 \\ K \\ \end{array}\right] \\ \ ={}&-\Big [K^{\top }M^{-1}(\beta {}M+D_{u})M^{-1}K+(M+D_{y})\Big ]. \end{aligned}$$

Let us consider a ‘matching’ strategy once again, and write for our approximation:where ${\widehat{M}}_{2}$ is selected to incorporate the second term of *S*, i.e.which may be achieved if

$$\begin{aligned} {\widehat{M}}_{2}\approx (M+D_{y})^{1/2}(\beta {}M+D_{u})^{-1/2}M. \end{aligned}$$

For a practical preconditioner, we in fact select

$$\begin{aligned} {\widehat{M}}_{2}=\big [\text {diag}(M+D_{y})\big ]^{1/2}\big [\text {diag} (\beta {}M+D_{u})\big ]^{-1/2}M. \end{aligned}$$

To approximate $K^{\top }+{\widehat{M}}_{2}$ and  in practice, we again make use of the AGMG software to apply a multigrid process to the relevant matrices within ${\widehat{S}}_{2}$.

One may therefore build a block triangular preconditioner for the permuted system (41), of the form ${\mathcal {P}}_{T}$ in (31). Rearranging the matrix system (and hence the preconditioner) to the form (24), we are therefore able to construct the following preconditioner for (24):

$$\begin{aligned} {\mathcal {P}}_{3}=\left[ \begin{array}{c@{\quad }c@{\quad }c} -{\widehat{S}}_{2} &{} 0 &{} K^{\top } \\ 0 &{} \beta {}M+D_{u} &{} -M_{\text {cheb}} \\ 0 &{} -M_{\text {cheb}} &{} 0 \\ \end{array}\right] , \end{aligned}$$

where $M_{\text {cheb}}$ relates to a Chebyshev semi-iteration process for the mass matrix *M*. We notice that this relates to observations made on nullspace preconditioners for saddle point systems in [38].

It is clear that to apply the preconditioner ${\mathcal {P}}_{3}$, we require a non-symmetric solver such as Gmres, as it is not possible to construct a positive definite preconditioner with this rearrangement of the matrix system. Within such a solver, a key positive property of this strategy is that we may approximate $\varPhi$ almost perfectly (and cheaply), and may apply $K^{\top }$ exactly within ${\mathcal {P}}_{3}$ without a matrix inversion. An associated disadvantage is that our approximation of *S* is more expensive to apply than the approximation ${\widehat{S}}_{1}$ used within the preconditioners ${\mathcal {P}}_{1}$ and ${\mathcal {P}}_{2}$—whereas Theorem 1 may again be applied^4^ to verify a lower bound for the eigenvalues of the preconditioned Schur complement, the values of the largest eigenvalues are frequently found to be higher than for the Schur complement approximation ${\widehat{S}}_{1}$ described earlier.

### Time-dependent problems

Due to the huge dimensions of the matrix systems arising from time-dependent PDE-constrained optimization problems, it is very important to consider preconditioners for the resulting systems, which are of the form (29). These are again of saddle point type (30), with

$$\begin{aligned} \ \varPhi =\left[ \begin{array}{c@{\quad }c} \tau {\mathcal {M}}_{1/2}+{\mathcal {D}}_{y} &{} 0 \\ 0 &{} \beta \tau {\mathcal {M}}_{1/2}+{\mathcal {D}}_{u} \\ \end{array}\right] ,\quad \quad \varPsi =\left[ \begin{array}{c@{\quad }c} {\mathcal {K}} &{} -\tau {\mathcal {M}} \\ \end{array}\right] ,\quad \quad \varTheta =\left[ \begin{array}{c} 0 \\ \end{array}\right] . \end{aligned}$$

As for the time-independent case we may approximate $\varPhi$ using diagonal solves or the Chebyshev semi-iteration method applied to the matrices from each time-step.

To approximate the Schur complement of (29),

$$\begin{aligned} \mathcal {S}={\mathcal {K}}\big (\tau {\mathcal {M}}_{1/2}+{\mathcal {D}}_{y} \big )^{-1}{\mathcal {K}}^{\top }+\tau ^{2}{\mathcal {M}}\big (\beta \tau {\mathcal {M}}_{1/2}+{\mathcal {D}}_{u}\big )^{-1}{\mathcal {M}}, \end{aligned}$$

we again apply a matching strategy to obtain

$$\begin{aligned} {\widehat{\mathcal {S}}}_{1,T}:=\big ({\mathcal {K}}+{\widehat{{\mathcal {M}}}}_{1,T} \big )\big (\tau {\mathcal {M}}_{1/2}+{\mathcal {D}}_{y}\big )^{-1}\big ({\mathcal {K}} +{\widehat{{\mathcal {M}}}}_{1,T}\big )^{\top }, \end{aligned}$$

whereThis in turn motivates the choice

$$\begin{aligned} {\widehat{{\mathcal {M}}}}_{1,T}=\tau {\mathcal {M}}\left[ \text {diag} \big (\beta \tau {\mathcal {M}}_{1/2}+{\mathcal {D}}_{u}\big )\right] ^{-1/2} \left[ \text {diag}\big (\tau {\mathcal {M}}_{1/2}+{\mathcal {D}}_{y}\big )\right] ^{1/2}, \end{aligned}$$

and we require two multigrid processes per time-step to apply ${\widehat{\mathcal {S}}}_{1,T}^{-1}$ efficiently.

Combining our approximations of (1, 1)-block and Schur complement, we may apply

$$\begin{aligned} {\mathcal {P}}_{1,T}=\left[ \begin{array}{c@{\quad }c@{\quad }c} \big (\tau {\mathcal {M}}_{1/2}+{\mathcal {D}}_{y}\big )_{\text {approx}} &{} 0 &{} 0 \\ 0 &{} \big (\beta \tau {\mathcal {M}}_{1/2}+{\mathcal {D}}_{u}\big )_{\text {approx}} &{} 0 \\ 0 &{} 0 &{} {\widehat{\mathcal {S}}}_{1,T} \\ \end{array}\right] \end{aligned}$$

within Minres, for example, or

$$\begin{aligned} {\mathcal {P}}_{2,T}=\left[ \begin{array}{c@{\quad }c@{\quad }c} \big (\tau {\mathcal {M}}_{1/2}+{\mathcal {D}}_{y}\big )_{\text {approx}} &{} 0 &{} 0 \\ 0 &{} \big (\beta \tau {\mathcal {M}}_{1/2}+{\mathcal {D}}_{u}\big )_{\text {approx}} &{} 0 \\ {\mathcal {K}} &{} -\tau {\mathcal {M}} &{} -{\widehat{\mathcal {S}}}_{1,T} \\ \end{array}\right] , \end{aligned}$$

within a nonsymmetric solver such as Gmres.

Alternatively, in complete analogy to the time-independent setting, one could rearrange the matrix system such that the (1, 1)-block may be approximated accurately, and select the preconditioner

$$\begin{aligned} {\mathcal {P}}_{3,T}=\left[ \begin{array}{c@{\quad }c@{\quad }c} -{\widehat{\mathcal {S}}}_{2,T} &{} 0 &{} {\mathcal {K}}^{\top } \\ 0 &{} \beta \tau {\mathcal {M}}_{1/2}+{\mathcal {D}}_{u} &{} -\tau {\mathcal {M}}_{\text {cheb}} \\ 0 &{} -\tau {\mathcal {M}}_{\text {cheb}} &{} 0 \\ \end{array}\right] . \end{aligned}$$

Inverting ${\mathcal {M}}_{\text {cheb}}$ requires the application of Chebyshev semi-iteration to $N_{t}$ mass matrices *M*, and the Schur complement approximation is given bywith

$$\begin{aligned} \mathcal {{\widehat{M}}}_{2,T}=\tau \left[ \text {diag}\big (\tau {\mathcal {M}}_{1/2} +{\mathcal {D}}_{y}\big )\right] ^{1/2}\left[ \text {diag} \big (\beta \tau {\mathcal {M}}_{1/2}+{\mathcal {D}}_{u}\big )\right] ^{-1/2}{\mathcal {M}}. \end{aligned}$$

Similar eigenvalue results can be shown for the Schur complement approximation ${\widehat{\mathcal {S}}}_{1,T}$ as for the approximations used in the time-independent case.

#### Remark 1

We highlight that a class of methods which is frequently utilized when solving PDE-constrained optimization problems, aside from the iterative methods discussed in this paper, is that of multigrid. We recommend [8] for an overview of such methods for PDE-constrained optimization, [7] for a convergence analysis of multigrid applied to these problems, [20, 21] for schemes derived for solving flow control problems, and [6] for a method tailored to problems with additional bound constraints. These solvers require the careful construction of prolongation/restriction operators, as well as smoothing methods, tailored to the precise problem at hand. Applying multigrid to the entire coupled matrix systems resulting from the problems considered in this paper, as opposed to employing this technology to solve sub-blocks of the system within an iterative method, also has the potential to be a powerful approach for solving problems with bound constraints. Similar multigrid methods have previously been applied to the interior point solution of PDE-constrained optimization problems in one article [9], and we believe that a carefully tailored scheme could be a viable alternative when solving at least some of the numerical examples considered in Sect. 5.

### Alternative problem formulations

We have sought to illustrate our interior point solvers, and in particular the preconditioned iterative methods for the solution of the associated Newton systems, using quadratic tracking functionals with a quadratic cost for the control, as in (2). We now wish to briefly outline some of the possible extensions to this problem that we believe we could apply our method to, as below:

- *Boundary control problems*  Our methodology could be readily extended to problems where the control (or state) variable is regularized on the boundary only within the cost functional, for instance where
  $$\begin{aligned} {\mathcal {J}}(y,u)=\frac{1}{2} \Vert y - {\widehat{y}} \Vert _{L_{2}(\varOmega )}^2 + \frac{\beta }{2} \Vert u \Vert _{L_{2}(\partial \varOmega )}^2. \end{aligned}$$
  For such problems, we would need to take account of boundary mass matrices within the saddle point system that arises, however preconditioners have previously been designed for such problems that take into account these features (see [35], for instance).
- *Control variable regularized on a subdomain*  Analogously, problems may be considered using our preconditioning approach where the cost functional is of the form
  $$\begin{aligned} {\mathcal {J}}(y,u)=\frac{1}{2} \Vert y - {\widehat{y}} \Vert _{L_{2}(\varOmega )}^2 + \frac{\beta }{2} \Vert u \Vert _{L_{2}(\varOmega _1)}^2, \end{aligned}$$
  where $\varOmega _1\subset \varOmega$. The matching strategy of Sect. 4.1 may be modified to account for the matrices of differing structures.
- *Alternative regularizations*  A further possibility is for the control (or state) variable to be regularized using a different term, for instance an $H^{1}$ regularization term of the following form:
  $$\begin{aligned} {\mathcal {J}}(y,u)= & {} \frac{1}{2} \Vert y - {\widehat{y}} \Vert _{L_{2}(\varOmega )}^2 + \frac{\beta }{2} \Vert u \Vert _{H^{1}(\varOmega )}^2\\= & {} \frac{1}{2} \Vert y - {\widehat{y}} \Vert _{L_{2}(\varOmega )}^2 + \frac{\beta }{2} \Vert u \Vert _{L_{2}(\varOmega )}^2 + \frac{\beta }{2} \Vert \nabla {}u \Vert _{L_{2}(\varOmega )}^2. \end{aligned}$$
  Upon discretization, stiffness matrices arise within the (1, 1)-block in addition to mass matrices, however the preconditioning method introduced in this paper may still be applied, by accounting for the new matrices within the matching strategy for the Schur complement.
- *Time-dependent problems*  Finally, we highlight that modifications to the cost functional considered for time-dependent problems in Sect. 3.3 may be made. For instance, one may measure the control (or state) variables at the final time only, that is
  $$\begin{aligned} {\mathcal {J}}(y,u)=\frac{1}{2}\int _{0}^{T}\int _{\varOmega }\big (y({\mathbf {x}},t) -{\widehat{y}}({\mathbf {x}},t)\big )^{2}~\mathrm{d}\varOmega \mathrm{d}t+\frac{\beta }{2}\int _{\varOmega }u({\mathbf {x}},T)^{2}~\mathrm{d}\varOmega . \end{aligned}$$
  On the discrete level, this will lead to mass matrices being removed from portions of the (1, 1)-block, and this information may be built into new preconditioners [35, 44].

We emphasize that there are some examples of cost functional, for instance a functional where a curl function is applied to state or control, or one which includes terms of the form $\int \max \{0,\text {det}(\nabla {}y)\}$ (see [19]), where the preconditioning approach presented here would not be directly applicable. As PDE-constrained optimization problems are widespread and varied in type, much useful further work could be carried out on extending the method presented in this paper to more diverse classes of optimization problems.

## Numerical experiments

Having motivated our numerical methods for the solution of the problems considered, we now wish to test our solvers on a range of examples. These test problems are of both time-independent and time-dependent form, and are solved on a desktop with a quad-core 3.2GHz processor. For each test problem, we discretize the state, control and adjoint variables using *Q*1 finite elements. Within the interior point method, the value of the barrier reduction parameter $\sigma$ is set to be 0.1, with $\alpha _{0}=0.995$, and $\epsilon _{p}=\epsilon _{d}=\epsilon _{c}=10^{-6}$. To solve the Newton systems arising from the interior point method, we use the Ifiss software package [11, 43] to construct the relevant finite element matrices. When the symmetric block diagonal preconditioner ${\mathcal {P}}_{1}$ is used, we solve the Newton systems using the Minres algorithm to a relative preconditioned residual norm tolerance of $10^{-8}$, and the Chebyshev semi-iteration method to approximate the inverse of the (1, 1)-block (apart from within one experiment where we use a diagonal approximation), as well as the AGMG method to approximate the inverse Schur complement. Where the block triangular preconditioners ${\mathcal {P}}_{2}$ and ${\mathcal {P}}_{3}$ are applied, we solve the Newton systems with the preconditioned Gmres method to a tolerance of $10^{-8}$; we apply 20 steps of Chebyshev semi-iteration to approximate the (1, 1)-block, and once again utilize AGMG for the Schur complement approximations. We highlight that it would be feasible to relax the tolerances for Minres and Gmres in order to lower the overall CPU time for the interior point scheme [16], however we elect to solve the matrix systems relatively accurately in order to fully demonstrate the potency of our preconditioned iterative methods. All results are computed using Matlab R2015a.

**Fig. 1:**
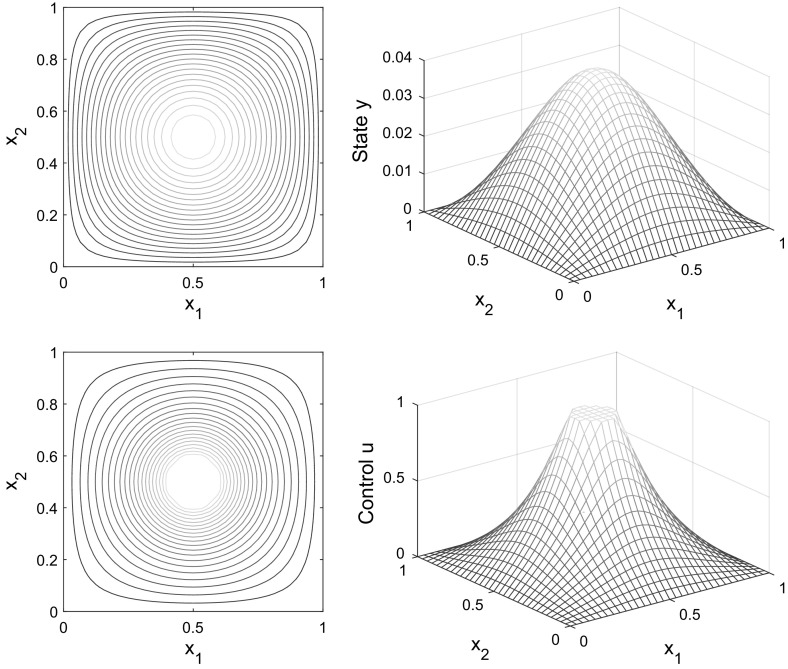
Contour and mesh plots of the solution to the Poisson control example with control constraints, for state variable *y* (*top*) and control variable *u* (*bottom*), with $\beta =10^{-2}$

*Control constrained problems*  The first experiments we carry out involve a Poisson control problem, with ${\mathcal {L}}=-\nabla ^{2}$ applied on $\varOmega :=[0,1]^{2}$, $y=0$ on the boundary of $\varOmega$, and the desired state given by ${\widehat{y}}=e^{-64\left( (x_{1}-0.5)^{2}+(x_{2}-0.5)^{2}\right) }$, where the spatial coordinates $\mathbf {x}=\left[ x_{1},~x_{2}\right] ^{\top }$. We solve this problem using the Minres algorithm with preconditioner ${\mathcal {P}}_{1}$, using both the Chebyshev semi-iteration method and the matrix diagonal to approximate the (1, 1)-block within the preconditioner. The results obtained are shown in Table 1, for a range of mesh-sizes *h* and regularization parameters $\beta$. A solution plot for $\beta =10^{-2}$ is also shown in Fig. 1. We select box constraints for the control variable only, based on the value of $\beta$ used and the behaviour of the optimal control problem when no bound constraints are imposed—we are careful to make sure that the constraints are sensible physically, but also challenging for our interior point solver. The constraints taken for each value of $\beta$ are stated in Table 1. It is worth pointing out that increasing the accuracy of discretization (decreasing *h* by a factor of 2) typically adds about one extra interior point iteration, which once again demonstrates that interior point methods are not very sensitive to the problem dimension (as discussed in [17], for instance). We find that both the number of iterations of the interior point method, and the average number of Minres iterations per interior point (Newton) step, are very reasonable for the problem considered. Whereas we observe an increase in iterative steps for the more challenging case of smaller $\beta$, all numbers are low, in particular the very encouraging iteration counts for moderate regularization parameters. We also find that, as one might expect, the computational cheapness of a diagonal approximation of the (1, 1)-block is counteracted by the higher Minres iteration numbers that result.

**Table 1:** Results for the Poisson control example with control constraints, for a range of values of *h* and $\beta$, and preconditioner ${\mathcal {P}}_{1}$

	$\beta =1$		$\beta =10^{-1}$		$\beta =10^{-2}$		$\beta =10^{-3}$		$\beta =10^{-4}$		$\beta =10^{-5}$		$\beta =10^{-6}$	
	$u\ge 0$		$u\ge 0$		$u\ge 0$		$u\ge 0$		$u\ge 0$		$u\ge 0$		$u\ge 0$	
	$u\le 0.01$		$u\le 0.1$		$u\le 1$		$u\le 3$		$u\le 20$		$u\le 100$		$u\le 300$	
${\mathcal {P}}_{1}$ Chebyshev														
*h*														
$2^{-2}$	*10*	5.6	*11*	6.3	*13*	6.2	*15*	6.6	*18*	7.5	*19*	7.2	*20*	7.4
$2^{-3}$	*10*	5.7	*13*	6.1	*14*	6.3	*16*	7.8	*19*	8.3	*20*	8.7	*21*	9.3
$2^{-4}$	*10*	5.6	*13*	6.1	*15*	6.5	*19*	7.4	*22*	8.6	*22*	8.5	*21*	8.8
$2^{-5}$	*11*	5.4	*16*	5.8	*18*	6.3	*21*	7.0	*23*	8.8	*25*	8.9	*24*	9.4
$2^{-6}$	*11*	5.5	*16*	5.8	*20*	6.2	*22*	6.8	*26*	15.5	*24*	8.9	*30*	9.4
$2^{-7}$	*12*	5.2	*18*	5.5	*20*	6.2	*20*	7.1	*27*	8.4	*25*	8.6	*31*	9.2
${\mathcal {P}}_{1}$ Diagonal														
*h*														
$2^{-2}$	*9*	9.4	*11*	10.4	*13*	9.5	*15*	9.2	*18*	10.1	*19*	9.4	*20*	17.6
$2^{-3}$	*10*	15.1	*12*	16.7	*14*	16.9	*16*	18.4	*19*	17.5	*20*	18.5	*21*	19.5
$2^{-4}$	*10*	15.5	*15*	18.6	*16*	19.9	*19*	22.7	*22*	21.6	*22*	23.4	*21*	24.3
$2^{-5}$	*11*	16.3	*16*	16.2	*19*	19.5	*21*	21.1	*23*	24.7	*25*	25.7	*24*	25.8
$2^{-6}$	*11*	15.5	*16*	20.2	*20*	16.9	*22*	18.9	*26*	32.1	*24*	18.9	*31*	26.7
$2^{-7}$	*12*	14.3	*18*	15.7	*21*	16.1	*20*	18.5	*28*	28.8	*25*	19.3	*31*	23.4

Presented are the number of interior point (Newton) iterations required to achieve convergence (italic, left), and average number of Minres steps per interior point iteration before a relative preconditioned residual norm of $10^{-8}$ is achieved (plain text, right). Results are given with a Chebyshev semi-iteration method applied to the (1, 1)-block (top), and with a diagonal approximation (bottom)

*Problems with state constraints* We next examine a Poisson control problem involving state constraints, where , and $y={\widehat{y}}$ on the boundary of $\varOmega$. We apply the preconditioners ${\mathcal {P}}_{2}$ (with Chebyshev semi-iteration used to approximate the (1, 1)-block) and ${\mathcal {P}}_{3}$, and solve using Gmres to a tolerance of $10^{-8}$ for a range of *h* and $\beta$. Again the results, which are presented in Table 2, are very promising when either preconditioner is used, and a large degree of robustness is achieved despite the very general matrix systems which can arise at each interior point iteration. We highlight that the iteration counts are likely to vary depending on how severe the box constraints that we impose are, as the structure of the matrices can change drastically. In Table 3 we present results for this problem (for $\beta =10^{-2}$) with preconditioners ${\mathcal {P}}_{1}$, ${\mathcal {P}}_{2}$ and ${\mathcal {P}}_{3}$—we observe in particular that the CPU times scale in an approximately linear fashion with the dimension of the matrix systems being solved.

**Table 2:** Results for the Poisson control example with state constraints, for a range of values of *h* and $\beta$

	$\beta =1$		$\beta =10^{-2}$		$\beta =10^{-4}$		$\beta =10^{-6}$	
	$-0.1\le {}y\le 0.002$		$-0.1\le {}y\le 0.175$		$-0.1\le {}y\le 0.9$		$-0.1\le {}y\le 1$	
$${\mathcal {P}}_{2}$$								
*h*								
$2^{-2}$	*11*	5.3	*8*	5.0	*9*	5.0	*10*	5.0
$2^{-3}$	*12*	9.9	*9*	10.2	*10*	13.3	*10*	10.9
$2^{-4}$	*13*	11.4	*10*	12.9	*11*	16.8	*11*	13.5
$2^{-5}$	*14*	12.1	*11*	13.3	*13*	27.4	*12*	15.0
$2^{-6}$	*16*	12.5	*12*	13.6	*14*	17.8	*13*	15.7
$2^{-7}$	*17*	12.7	*13*	14.6	*16*	16.9	*14*	16.3
$${\mathcal {P}}_{3}$$								
*h*								
$2^{-2}$	*11*	5.0	*8*	5.1	*9*	5.0	*10*	5.0
$2^{-3}$	*12*	9.6	*9*	9.1	*10*	10.5	*10*	10.5
$2^{-4}$	*13*	11.2	*10*	10.3	*11*	12.3	*11*	12.4
$2^{-5}$	*14*	12.1	*11*	10.8	*13*	12.9	*12*	13.5
$2^{-6}$	*16*	12.6	*12*	11.4	*14*	13.3	*13*	13.9
$2^{-7}$	*17*	13.1	*13*	13.0	*16*	13.5	*14*	14.5

Presented are the number of interior point iterations required to achieve convergence (italic, left), and average number of Gmres steps needed (plain text, right). Results are given when the preconditioners ${\mathcal {P}}_{2}$ (top) and ${\mathcal {P}}_{3}$ (bottom) are used

**Table 3:** Number of interior point (Newton) iterations, average number of iterations of the Krylov subspace method per interior point step, and CPU time required to solve the Poisson control example with state constraints, when the preconditioners ${\mathcal {P}}_{1}$, ${\mathcal {P}}_{2}$ and ${\mathcal {P}}_{3}$ are used

$\beta =10^{-2}$	${\mathcal {P}}_{1}$			${\mathcal {P}}_{2}$			${\mathcal {P}}_{3}$		
	IPM	Krylov	CPU	IPM	Krylov	CPU	IPM	Krylov	CPU
*h*									
$2^{-2}$	*8*	8.0	**0**.**13**	*8*	5.0	**0**.**20**	*8*	5.1	**0**.**22**
$2^{-3}$	*9*	11.8	**0**.**23**	*9*	10.2	**0**.**35**	*9*	9.1	**0**.**34**
$2^{-4}$	*10*	14.5	**0**.**46**	*10*	12.9	**0**.**63**	*10*	10.3	**0**.**57**
$2^{-5}$	*11*	14.1	**1**.**8**	*11*	13.3	**2**.**6**	*11*	10.8	**2**.**4**
$2^{-6}$	*13*	14.8	**9**.**1**	*12*	13.6	**11**.**4**	*12*	11.4	**10**.**1**
$2^{-7}$	*14*	14.9	**37**.**4**	*13*	14.6	**54**.**4**	*13*	13.0	**53**.**8**

Results are presented for a range of *h*, and fixed $\beta =10^{-2}$

Number of interior point (Newton) iterations is shown in italics. CPU time required to solve the Poisson control example with state constraints is shown in bold

In order to illustrate the potential of our solvers to handle PDE constraints of varying forms, in Table 4 we present results where the PDE constraint is an indefinite Helmholtz equation, that is ${\mathcal {L}}y=-\nabla ^{2}y-k^{2}y$ for a given (positive) parameter *k*. We highlight that the forward Helmholtz equation itself is a notoriously difficult problem to solve numerically [12], and a great deal of research has been undertaken concerning the preconditioning of such systems (we recommend [13] for a discussion of shifted Laplacian preconditioners for these problems). We therefore emphasize that, given the challenges involved and the inherent indefiniteness of the problem, it is extremely difficult to obtain completely robust solvers, and much future research could be undertaken in this area. However the results obtained indicate that, at least for some test problems, the interior point method presented can be applied for a range of parameter setups.

**Table 4:** Results for the Helmholtz problem with state constraints, for a range of values of *h* and $\beta$, as well as values of *k*

	$k=20$						$k=50$			
	$\beta =10^{-2}$		$\beta =10^{-4}$		$\beta =10^{-6}$		$\beta =10^{-2}$		$\beta =10^{-4}$	
	$-0.0005\le {}y\le 0.0005$		$-0.05\le {}y\le 0.05$		$-0.6\le {}y\le 0.6$		$-10^{-5}\le {}y\le 10^{-5}$		$-0.001\le {}y\le 0.001$	
$${\mathcal {P}}_{2}$$										
*h*										
$2^{-2}$	*7*	4.3	*10*	5.3	*10*	4.7	*5*	4.0	*8*	4.7
$2^{-3}$	*8*	9.2	*10*	11.6	*11*	12.4	*5*	6.8	*8*	12.3
$2^{-4}$	*8*	10.6	*11*	17.7	*12*	30.8	*6*	10.4	*8*	17.9
$2^{-5}$	*9*	11.2	*12*	18.8	*12*	19.6	*6*	6.1	*9*	20.5
$2^{-6}$	*9*	10.4	*12*	15.9	*13*	22.7	*7*	10.3	*10*	23.1
$2^{-7}$	*10*	10.2	*13*	15.6	*14*	15.5	*8*	10.6	*10*	20.1
$${\mathcal {P}}_{3}$$										
*h*										
$2^{-2}$	*7*	4.2	*10*	5.2	*10*	3.9	*5*	3.7	*8*	5.1
$2^{-3}$	*8*	9.3	*10*	11.8	*11*	8.2	*5*	6.2	*8*	10.4
$2^{-4}$	*8*	9.9	*11*	13.9	*12*	10.0	*6*	9.3	*8*	16.8
$2^{-5}$	*9*	10.9	*12*	15.2	*12*	10.2	*6*	5.1	*9*	19.1
$2^{-6}$	*9*	10.2	*12*	15.1	*13*	10.4	*7*	9.5	*10*	21.8
$2^{-7}$	*10*	10.3	*13*	14.7	*14*	10.2	*8*	10.0	*10*	18.5

Presented are the number of interior point iterations required to achieve convergence (italic, left), and average number of Gmres steps needed (plain text, right). Results are given when the preconditioners ${\mathcal {P}}_{2}$ (top) and ${\mathcal {P}}_{3}$ (bottom) are used

**Fig. 2:**
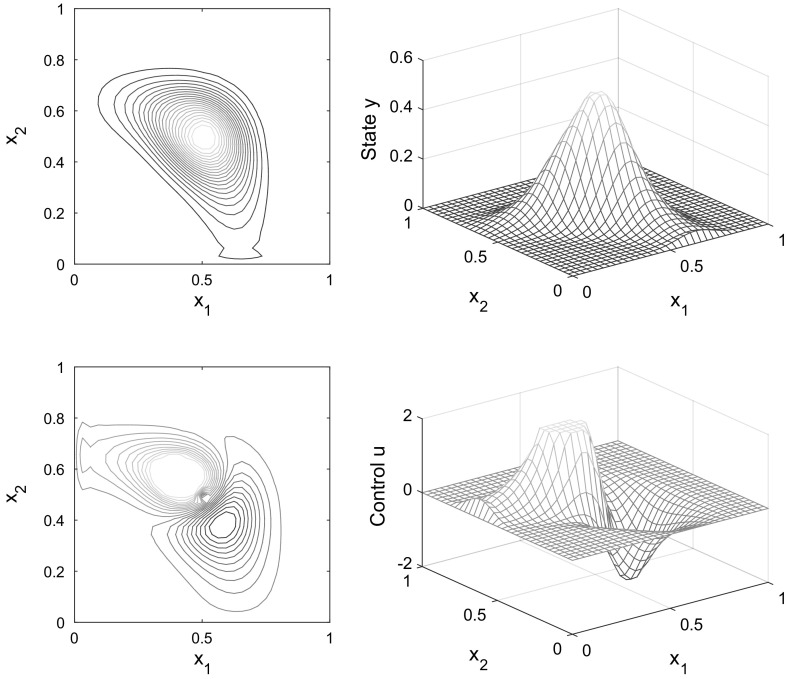
Contour and mesh plots of the solution to the convection-diffusion control example with state and control constraints, for state variable *y* (*top*) and control variable *u* (*bottom*), with $\beta =10^{-2}$

**Table 5:** Results for the convection-diffusion control example with state and control constraints, for a range of values of *h* and $\beta$

	$\beta =10^{-1}$		$\beta =10^{-2}$		$\beta =10^{-3}$		$\beta =10^{-4}$		$\beta =10^{-5}$	
	$0\le {}y\le 0.2$		$0\le {}y\le 0.5$		$0\le {}y\le 0.5$		$0\le {}y\le 0.75$		$0\le {}y\le 0.75$	
	$-0.75\le {}u\le 0.75$		$-2\le {}u\le 2$		$-3\le {}u\le 3$		$-5\le {}u\le 5$		$-6\le {}u\le 6$	
$${\mathcal {P}}_{2}$$										
*h*										
$2^{-2}$	*13*	10.1	*14*	11.3	*14*	11.3	*14*	11.1	*14*	11.4
$2^{-3}$	*14*	20.9	*15*	19.8	*15*	24.8	*15*	21.8	*15*	22.4
$2^{-4}$	*16*	35.1	*15*	20.6	*16*	42.6	*16*	37.6	*17*	53.0
$2^{-5}$	*17*	44.1	*17*	40.4	*16*	45.6	*19*	64.3	*19*	69.3
$2^{-6}$	*19*	52.6	*19*	48.4	*17*	47.3	*22*	66.7	*23*	73.6
$2^{-7}$	*21*	50.0	*21*	48.2	*22*	63.5	*26*	75.0	*27*	81.7
$${\mathcal {P}}_{3}$$										
*h*										
$2^{-2}$	*13*	8.9	*14*	9.1	*15*	9.5	*14*	8.7	*14*	8.8
$2^{-3}$	*14*	11.3	*15*	10.9	*15*	12.1	*15*	12.2	*15*	11.8
$2^{-4}$	*15*	13.1	*15*	11.8	*16*	13.4	*16*	13.3	*16*	14.1
$2^{-5}$	*17*	13.9	*17*	13.3	*16*	14.7	*19*	13.7	*19*	14.9
$2^{-6}$	*19*	14.6	*19*	14.5	*17*	17.9	*22*	16.6	*23*	16.3
$2^{-7}$	*21*	23.0	*21*	14.9	*22*	17.3	*26*	17.7	*27*	18.4

Presented are the number of interior point iterations required to achieve convergence (italic, left), and average number of Gmres steps needed (plain text, right). Results are given when the preconditioners ${\mathcal {P}}_{2}$ (top) and ${\mathcal {P}}_{3}$ (bottom) are used

*Both state and control constraints* In Table 5 we investigate a problem of convection-diffusion control type, with ${\mathcal {L}}=-0.01\nabla ^{2}+\big [-\frac{1}{\sqrt{2}},~\frac{1}{\sqrt{2}}\big ]^{\top }\cdot \nabla$, and ${\widehat{y}}=e^{-64\left( (x_{1}-0.5)^{2}+(x_{2}-0.5)^{2}\right) }$. We now impose both state and control constraints (as specified for each value of $\beta$), and test the preconditioners ${\mathcal {P}}_{2}$ and ${\mathcal {P}}_{3}$ using Gmres. We also present a solution plot for $\beta =10^{-2}$ in Fig. 2. For convection-diffusion control problems such as this, we find there is a great advantage in applying the preconditioner ${\mathcal {P}}_{3}$ over the preconditioner ${\mathcal {P}}_{2}$, due in part to the accurate approximation of the (1, 1)-block within it. Indeed this is demonstrated by the numbers of Gmres iterations required, which are much lower when using the preconditioner ${\mathcal {P}}_{3}$, especially for the final interior point iterations when convergence is close to being achieved. The Gmres solver with ${\mathcal {P}}_{3}$ demonstrates excellent robustness considering the complexity of the problem.

**Table 6:** Results for the three dimensional Poisson control example with control constraints, for a range of values of *h* and $\beta$, and preconditioner ${\mathcal {P}}_{1}$

${\mathcal {P}}_{1}$ Chebyshev	$\beta =1$		$\beta =10^{-1}$		$\beta =10^{-2}$		$\beta =10^{-3}$		$\beta =10^{-4}$		$\beta =10^{-5}$		$\beta =10^{-6}$	
	$u\ge 0$		$u\ge 0$		$u\ge 0$		$u\ge 0$		$u\ge 0$		$u\ge 0$		$u\ge 0$	
	$u\le 0.01$		$u\le 0.1$		$u\le 1$		$u\le 3$		$u\le 20$		$u\le 100$		$u\le 300$	
*h*														
$2^{-2}$	*7*	10.8	*8*	10.7	*9*	11.1	*10*	11.2	*11*	11.2	*12*	11.1	*12*	11.4
$2^{-3}$	*8*	10.7	*9*	10.8	*11*	10.4	*11*	11.0	*12*	11.0	*13*	11.1	*12*	11.1
$2^{-4}$	*9*	10.9	*10*	10.8	*11*	10.8	*12*	10.9	*12*	11.2	*13*	11.1	*13*	11.1
$2^{-5}$	*11*	14.1	*12*	14.0	*12*	13.8	*13*	13.8	*13*	13.6	*14*	13.5	*14*	13.8

Presented are the number of interior point (Newton) iterations required to achieve convergence (italic, left), and average number of Minres steps per interior point iteration before a relative preconditioned residual norm of $10^{-8}$ is achieved (plain text, right)

*3D test problems* It is also important to emphasize that the methodology presented in this work can be readily applied to three dimensional test problems—indeed these are problems for which it is generally accepted that preconditioned iterative methods are essential, as the huge computer storage requirements associated with such problems ensure that direct methods are out of reach. We therefore experiment using a Poisson control problem applied on the domain $\varOmega :=[0,1]^{3}$, with desired state ${\widehat{y}}=e^{-64\left( (x_{1}-0.5)^{2}+(x_{2}-0.5)^{2}+(x_{3}-0.5)^{2}\right) }$ and spatial coordinates $\mathbf {x}=\left[ x_{1},~x_{2},~x_{3}\right] ^{\top }$. We present numerical results in Table 6, demonstrating that, as for two dimensional problems, rapid convergence is achieved with robustness in problem size and regularization parameter.

**Table 7:** Results for the heat equation control example with control constraints, for a range of values of *h*, $\tau$, and $\beta$, and preconditioner ${\mathcal {P}}_{1,T}$

	$\beta =10^{-1}$		$\beta =10^{-2}$		$\beta =10^{-3}$		$\beta =10^{-4}$	
	$0\le {}u\le 0.1$		$0\le {}u\le 1$		$0\le {}u\le 3$		$0\le {}u\le 30$	
$${\mathcal {P}}_{1,T} \,\, (\tau =0.04)$$								
*h*								
$2^{-2}$	*13*	13.1	*15*	16.5	*16*	19.7	*21*	31.3
$2^{-3}$	*15*	13.7	*16*	16.6	*18*	20.5	*24*	30.6
$2^{-4}$	*16*	14.0	*18*	17.1	*20*	20.8	*24*	28.2
$2^{-5}$	*16*	14.0	*19*	17.5	*21*	21.0	*25*	27.5
$2^{-6}$	*18*	14.5	*19*	17.5	*22*	21.1	*27*	27.2
$${\mathcal {P}}_{1,T} \,\, (\tau =0.02)$$								
*h*								
$2^{-2}$	*14*	13.0	*16*	15.9	*17*	19.8	*23*	31.9
$2^{-3}$	*15*	13.4	*17*	15.6	*19*	20.5	*25*	30.9
$2^{-4}$	*16*	13.7	*18*	16.0	*21*	20.9	*25*	27.6
$2^{-5}$	*17*	14.0	*19*	16.4	*22*	21.1	*28*	28.3
$2^{-6}$	*15*	13.4	*19*	16.2	*22*	20.8	*27*	27.6
$${\mathcal {P}}_{1,T} \,\, (\tau =0.01)$$								
*h*								
$2^{-2}$	*14*	12.2	*16*	15.4	*18*	19.6	*24*	31.0
$2^{-3}$	*15*	12.4	*18*	15.7	*19*	19.9	*28*	30.9
$2^{-4}$	*16*	12.8	*18*	15.7	*21*	20.2	*27*	28.2
$2^{-5}$	*16*	12.8	*18*	15.7	*22*	20.5	*30*	28.3
$2^{-6}$	*17*	13.0	*19*	15.8	*22*	20.4	*29*	28.5

Presented are the number of interior point iterations required to achieve convergence (italic, left), and average number of Minres steps needed (plain text, right)

**Table 8:** Results for the heat equation control example with control constraints, for a range of values of *h*, $\tau$, and $\beta$, and preconditioner ${\mathcal {P}}_{2,T}$

${\mathcal {P}}_{2,T}$	$\tau =0.04$								$\tau =0.02$							
	$\beta =10^{-1}$		$\beta =10^{-2}$		$\beta =10^{-3}$		$\beta =10^{-4}$		$\beta =10^{-1}$		$\beta =10^{-2}$		$\beta =10^{-3}$		$\beta =10^{-4}$	
	$0\le {}u\le 0.1$		$0\le {}u\le 1$		$0\le {}u\le 3$		$0\le {}u\le 30$		$0\le {}u\le 0.1$		$0\le {}u\le 1$		$0\le {}u\le 3$		$0\le {}u\le 30$	
*h*																
$2^{-2}$	*13*	8.1	*15*	9.9	*16*	11.7	*21*	18.1	*14*	7.9	*16*	9.6	*17*	11.8	*23*	18.5
$2^{-3}$	*15*	8.4	*16*	9.9	*18*	11.8	*24*	17.2	*15*	8.2	*17*	9.6	*19*	12.1	*25*	17.7
$2^{-4}$	*16*	8.5	*18*	10.3	*20*	12.1	*24*	16.3	*16*	8.4	*18*	9.8	*21*	12.4	*25*	16.2
$2^{-5}$	*16*	8.5	*19*	10.4	*21*	12.2	*25*	16.1	*17*	8.5	*19*	10.0	*22*	12.3	*28*	16.8
$2^{-6}$	*18*	8.8	*19*	10.4	*22*	12.7	*27*	16.3	*15*	8.2	*19*	9.9	*22*	12.6	*27*	16.5

Presented are the number of interior point iterations required to achieve convergence (italic, left), and average number of Gmres steps needed (plain text, right)

**Table 9:** Results for the wave equation example with control constraints, for a range of values of *h*, $\tau$, and $\beta$

	${\mathcal {P}}_{1,T},~~h=2^{-4}$			${\mathcal {P}}_{1,T},~~h=2^{-5}$			${\mathcal {P}}_{2,T},~~h=2^{-4}$			${\mathcal {P}}_{2,T},~~h=2^{-5}$		
	$\beta$			$\beta$			$\beta$			$\beta$		
	$10^{-2}$	$10^{-3}$	$10^{-4}$	$10^{-2}$	$10^{-3}$	$10^{-4}$	$10^{-2}$	$10^{-3}$	$10^{-4}$	$10^{-2}$	$10^{-3}$	$10^{-4}$
$$\tau $$												
0.04	13.7	17.7	13.3	14.7	18.0	13.4	10.1	12.1	9.9	11.1	12.7	10.0
0.02	12.5	12.5	13.1	11.7	12.7	13.1	8.9	9.2	9.9	8.6	9.1	9.8
0.01	14.7	10.9	10.6	31.6	50.8	10.9	10.9	13.5	8.1	22.8	23.7	8.5
0.005	26.9	29.2	30.7	37.3	42.9	35.7	21.5	22.2	24.0	21.8	22.5	22.1

Presented are the average number of Minres (with preconditioner ${\mathcal {P}}_{1,T}$) and Gmres (with preconditioner ${\mathcal {P}}_{2,T}$) iterations required to solve the Newton systems obtained

*Time-dependent PDE constraints* To demonstrate that our solvers are also able to handle matrix systems of vast dimension arising from time-dependent PDE-constrained optimization problems, we present results in Table 7 for a heat equation control problem, with the PDE constraint given by $y_{t}-\nabla ^{2}y=u$ (for $t\in (0,1]$), and with additional control constraints imposed. The number of interior point iterations, and average Minres iteration count when ${\mathcal {P}}_{1,T}$ is applied, are provided for a range of *h* and $\beta$. As mentioned earlier, the backward Euler method is used for the time discretization, and values of $\tau =0.04$, 0.02 and 0.01 are tested for the time-step (in other words with 25, 50 and 100 time intervals). In Table 8, we present results obtained for the same problem using block triangular preconditioner ${\mathcal {P}}_{2,T}$ with Gmres. We once again observe a high degree of robustness in problem size (whether increased by refining the mesh in the spatial coordinates, or by decreasing the time-step) and regularization parameter.

Our final investigation involves the optimal control of the wave equation, which is the same problem as above, except with the PDE operator $y_{tt}-\nabla ^{2}y=u$ and with an initial condition imposed on $y_{t}$ (which we set to be zero). The recent work [27] derives an implicit scheme for this problem, which involves averaging the Laplacian term in the PDE operator. Within the matrix ${\mathcal {K}}$, this leads to discrete approximations of the operator $I-\frac{\tau ^{2}}{2}\nabla ^{2}$ on the block diagonal entries, as well as additional entries on the two blocks below the diagonal (corresponding to the operators $-2I$ and $I-\frac{\tau ^{2}}{2}\nabla ^{2}$, respectively). The method is designed to be unconditionally convergent, while also removing the requirement of a Courant–Friedrichs–Lewy (CFL) condition of the form $\tau \le {}h$ [27]. We investigate the potency of our preconditioners for this matrix system. In Table 9, we present the average number of Minres or Gmres iterations required to solve the systems arising from the interior point method. Although there is a larger variation in the number of steps required, due to the additional terms within the matrix system, the performance of the method is very encouraging considering the high complexity of the problem. We emphasize once again that the performance of the method is dependent somewhat on the severity of the box constraints imposed, however the numerical results obtained for a range of time-independent and time-dependent PDE-constrained optimization problems demonstrate the potency of the solvers presented in this manuscript.

## Concluding remarks

In this paper we have presented a practical method for the interior point solution of a number of PDE-constrained optimization problems with state and control constraints, by reformulating the minimization of the discretized system as a quadratic programming problem. Having outlined the structure of the algorithm for solving these problems, we derived fast and feasible preconditioned iterative methods for solving the resulting Newton systems, which is the dominant portion of the algorithm in terms of computational work. Encouraging numerical results indicate the effectiveness and utility of our approach.

The problems we considered involved Poisson control, heat equation control, and both steady and time-dependent convection-diffusion control. A natural extension of this work would be to consider the control of systems of PDEs, for instance Stokes control and other problems in fluid flow, as well as the control of nonlinear PDEs, which arises in a wide range of practical scientific applications. The latter task would be accomplished by reformulating the discretization as a nonlinear programming problem—the robust solution of such formulations is a substantial challenge within the optimization community, but would represent significant progress in tackling real-world optimal control problems.

## Footnotes

1 For the problems considered in [39], bounds for $\alpha _{y}y+\alpha _{u}u$ are specified, where $\alpha _{y}$ and $\alpha _{u}$ are given constants.

2 This assumption holds for both Poisson control and convection-diffusion control problems, for instance.

3 The main assumption made is that ${\widehat{S}}_{G}$ is invertible. This certainly holds unless $({\bar{X}}+{\bar{Y}})^{\top }{\mathbf {v}}=\mathbf {0}$ for some ${\mathbf {v}}$, which in our setting implies that $M^{-1}(\beta {}M+D_{u})^{1/2}(M+D_{y})^{-1/2}K^{\top }$ has an eigenvalue exactly equal to $-1$. As the matrices *M*, $D_{y}$, $D_{u}$ and *K* are unlikely to interact closely at any Newton step, this is extremely unlikely to occur and our assumption is therefore reasonable.

4 In the notation of Theorem 1, the matrices involved are ${\bar{X}}=K^{\top }M^{-1}(\beta {}M+D_{u})^{1/2}$ and ${\bar{Y}}=(M+D_{y})^{1/2}$.
